# Supplementary figures and images for: Capsicum chinense Jacq. fruit plays an immunomodulatory role in cytokine attenuation and DNA damage protection
Source: PLoS One. 2025 Feb 25;20(2):e0319251. doi: 10.1371/journal.pone.0319251 (PMC11856283; doi:10.1371/journal.pone.0319251)

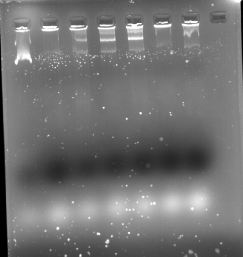

Supplement: S1 Fig — (JPG) [file pone.0319251.s001.jpg]

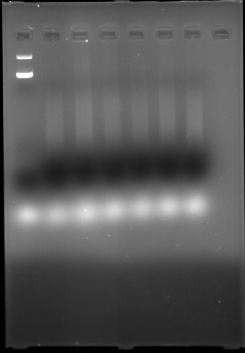

Supplement: S2 Fig — (JPG) [file pone.0319251.s002.jpg]

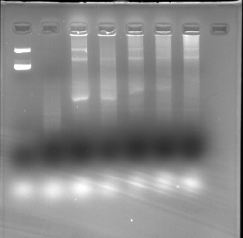

Supplement: S3 Fig — (JPG) [file pone.0319251.s003.jpg]
