# Supplementary material for: Capsicum chinense Jacq. fruit plays an immunomodulatory role in cytokine attenuation and DNA damage protection
Source: PLoS One. 2025 Feb 25;20(2):e0319251. doi: 10.1371/journal.pone.0319251 (PMC11856283; doi:10.1371/journal.pone.0319251)
Supplement: Attachment — Submitted filename: PONE-D-24-29500_reviewer.docx [file pone.0319251.s004.docx]

PLOS ONE

Capsicum chinense Jacq. fruit plays an immunomodulatory roles cytokine attenuation and DNA damage protection

--Manuscript Draft--

| Manuscript Number: | PONE-D-24-29500 |
| --- | --- |
| Article Type: | Research Article |
| Full Title: | Capsicum chinense Jacq. fruit plays an immunomodulatory role in cytokine attenuation and DNA damage protection |
| Short Title: | Immunomodulatory effects of Capsicum chinense Jacq |
| Corresponding Author: | Md. Atiar Rahman, Ph.D. University of Chittagong Chittagong, BANGLADESH |
| Keywords: | Antioxidants; Capsicum chinense Jacq.; DNA damage-protecting activity; cytotoxicity; anti-inflammatory; immunomodulatory |
| Abstract: | Capsicum chinense (C. chinense) Jacq. organic extracts (methanol, ethyl acetate and petroleum ether) have been investigated for their immunomodulatory and DNA-damage protecting potential. Immunomodulatory actions were undergone in SRBC-induced animal models connected with the evaluation of anti-inflammatory effects in LPS- stimulated RAW264.7 macrophage cells. Fenton reagent-based induction method was applied to measure the DNA damage protection effects. The results displayed that higher dosage of C. chinense methanol extract had a suppressive effect on the immunological response. The extracts minimized the pro-inflammatory mediators involving COX-2 and iNOS, and reduced the cytokines (TNF-α, IL-β, and IL-6) in LPS- stimulated RAW264.7 macrophage cells. The petroleum ether and methanol extracts were found to exhibit a more pronounced cytotoxic impact on the Hela cell line compared to the ethyl acetate extract. The higher dose of the extract was found to display a negative impact, while a lower dose reversed the immune reaction. The findings suggest that C. chinense extracts have the strong potential to regulate an innate immune system in animal models. |
| Order of Authors: | Md. Atiar Rahman, Ph.D. |
|  | Srabonti Saha, PhD |
|  | Md Altaf Hossain, MS |
|  | Humayra Ferdousy, MS |
|  | Jobaier ibne deen, MS |
|  | Akhlak Chowdhyry, MS |
|  | Md Mannan, MS |
|  | Md Asif Nadim Khan, MS |
| Additional Information: |  |
| Question | Response |
| Financial Disclosure  Enter a financial disclosure statement that describes the sources of funding for the work included in this submission. Review the [submission guidelines](http://journals.plos.org/plosone/s/submission-guidelines#loc-financial-disclosure-statement) for detailed requirements. View published research articles from [*PLOS ONE*](https://journals.plos.org/plosone/) for specific examples. | The author(s) received no specific funding for this work. |

| This statement is required for submission and will appear in the published article if the submission is accepted. Please make sure it is accurate. |  |
| --- | --- |
| Funded studies  Enter a statement with the following details:   - Initials of the authors who received each award - Grant numbers awarded to each author - The full name of each funder - URL of each funder website - Did the sponsors or funders play any role in the study design, data collection and analysis, decision to publish, or preparation of the manuscript? |  |
| Did you receive funding for this work? |  |
| Competing Interests  Use the instructions below to enter a competing interest statement for this submission. On behalf of all authors, disclose any [competing interests](http://journals.plos.org/plosone/s/competing-interests) that could be perceived to bias this work—acknowledging all financial support and any other relevant financial or non- financial competing interests.  This statement is required for submission and will appear in the published article if the submission is accepted. Please make sure it is accurate and that any funding sources listed in your Funding Information later in the submission form are also declared in your Financial Disclosure statement.  View published research articles from  [*PLOS ONE*](http://journals.plos.org/plosone/) for specific examples. | The authors have declared that no competing interests exist. |

| NO authors have competing interests  Enter: *The authors have declared that no competing interests exist.*  Authors with competing interests  Enter competing interest details beginning with this statement:  *I have read the journal's policy and the authors of this manuscript have the following competing interests: [insert competing interests here]* |  |
| --- | --- |
| * typeset |  |
| Ethics Statement  Enter an ethics statement for this submission. This statement is required if the study involved:   - Human participants - Human specimens or tissue - Vertebrate animals or cephalopods - Vertebrate embryos or tissues - Field research   Write "N/A" if the submission does not require an ethics statement.  General guidance is provided below. Consult the [submission guidelines](http://journals.plos.org/plosone/s/submission-guidelines#loc-guidelines-for-specific-study-types) for detailed instructions. Make sure that all information entered here is included in the Methods section of the manuscript. | Animal handling and cares were confirmed under an ethical guideline endorsed by the Institutional Ethical Committee of the Faculty of Biological Sciences of the University of Chittagong (Ethical approval no. AERB-FBSCU-20230605-(1) |

| Format for specific study types  Human Subject Research (involving human participants and/or tissue)   - Give the name of the institutional review board or ethics committee that approved the study - Include the approval number and/or a statement indicating approval of this research - Indicate the form of consent obtained (written/oral) or the reason that consent was not obtained (e.g. the data were analyzed anonymously)   Animal Research (involving vertebrate animals, embryos or tissues)   - Provide the name of the Institutional Animal Care and Use Committee (IACUC) or other relevant ethics board that reviewed the study protocol, and indicate whether they approved this research or granted a formal waiver of ethical approval - Include an approval number if one was obtained - If the study involved *non-human primates*, add *additional details* about animal welfare and steps taken to ameliorate suffering - If anesthesia, euthanasia, or any kind of animal sacrifice is part of the study, include briefly which substances and/or methods were applied   Field Research  Include the following details if this study involves the collection of plant, animal, or other materials from a natural setting:   - Field permit number - Name of the institution or relevant body that granted permission |  |
| --- | --- |
|  |  |
| Data Availability  Authors are required to make all data underlying the findings described fully available, without restriction, and from the time of publication. PLOS allows rare exceptions to address legal and ethical concerns. See the [PLOS Data Policy](http://journals.plos.org/plosone/s/data-availability) and [FAQ](http://journals.plos.org/plosone/s/data-availability#loc-faqs-for-data-policy) for detailed information. | Yes - all data are fully available without restriction |

| A Data Availability Statement describing where the data can be found is required at submission. Your answers to this question constitute the Data Availability Statement and will be published in the article, if accepted. |  |
| --- | --- |
| Important: Stating ‘data available on request from the author’ is not sufficient. If your data are only available upon request, select ‘No’ for the first question and explain your exceptional situation in the text box. |  |
| Do the authors confirm that all data underlying the findings described in their manuscript are fully available without restriction? |  |
| Describe where the data may be found in full sentences. If you are copying our sample text, replace any instances of XXX with the appropriate details.   - If the data are held or will be held in a public repository, include URLs, accession numbers or DOIs. If this information will only be available after acceptance, indicate this by ticking the box below. For example: *All XXX files are available from the XXX database (accession number(s) XXX, XXX.*). - If the data are all contained within the manuscript and/or Supporting Information files, enter the following: *All relevant data are within the manuscript and its Supporting Information files.* - If neither of these applies but you are able to provide details of access elsewhere, with or without limitations, please do so. For example:   *Data cannot be shared publicly because of [XXX]. Data are available from the XXX Institutional Data Access / Ethics Committee (contact via XXX) for researchers who meet the criteria for access to confidential data.*  *The data underlying the results presented in the study are available from (include the name of the third party* | Data will be available upon request |

| *and contact information or URL).*   - This text is appropriate if the data are owned by a third party and authors do not have permission to share the data.   * typeset |  |
| --- | --- |
| Additional data availability information: |  |

<https://www2.cloud.editorialmanager.com/pone/download.aspx?id=36933264&guid=ff724d3e-b239-4716-91a0-463f929b6b27&scheme=1>Manuscript	[Click here to](https://www2.cloud.editorialmanager.com/pone/download.aspx?id=36933264&guid=ff724d3e-b239-4716-91a0-463f929b6b27&scheme=1)

[access/download;Manuscript;Srabonti_manuscript_-](https://www2.cloud.editorialmanager.com/pone/download.aspx?id=36933264&guid=ff724d3e-b239-4716-91a0-463f929b6b27&scheme=1)

# 1 Full Length article

2 ***Capsicum chinense* Jacq. fruit plays an immunomodulatory roles cytokine attenuation and**

# 3 DNA damage protection

4

5 Srabonti Saha^1^, Md. Altaf Hossain^1^, Humayra Ferdousi^1^, Jobaier Ibne Deen^1^, Akhlak Chowdhury^1^,
6 Md. Mannan^1^, Md. Asif Nadim Khan^1^, Md. Atiar Rahman^1^*

7

8 ^1^Department of Biochemistry and Molecular Biology, University of Chittagong, Chittagong-
9 4331, Bangladesh.
10 ^2^Department of Genetic Engineering and Biotechnology, University of Chittagong, Chittagong-
11 4331, Bangladesh.

12

13	**Running title:** Immunomodulatory effects of *Capsicum chinense* Jacq.

14

15 **Corresponding author:** Md. Atiar Rahman Ph.D., Professor, Department of Biochemistry &
16 Molecular Biology, University of Chittagong, Chittagong-4331, Bangladesh, Tel: +88-031-
17 2606001-10, Extension- 4334, Fax: +88-031-726310, E-mail: [atiar@cu.ac.bd](mailto:atiar@cu.ac.bd)

18

19 **Email	of	the	authors:	Srabonti	Saha**	[**srabonti7@cu.ac.bd**](mailto:srabonti7@cu.ac.bd)**,	Md.	Altaf	Hossain-**
20 [**altafhossainanindo@gmail.com,**](mailto:altafhossainanindo@gmail.com) **Humayra Ferdousi-** [**ferdousihumayra@gmail.com,**](mailto:ferdousihumayra@gmail.com) **Jobaier**
21 **Ibne Deen-** [**jobaier.bmb@gmail.com,**](mailto:jobaier.bmb@gmail.com) **Akhlak** [**Chowdhury- akhlakchyemon@gmail.com**](mailto:Chowdhury-atiarh@yahoo.com)**;**
22 **Md.	Mannan-**	[**mdmannan956956@gmail.com,**](mailto:mdmannan956956@gmail.com)	**Md.	Asif	Nadim**	[**Khan-**](mailto:Khan-asifnadimkhan@gmail.com)
23 [**asifnadimkhan@gmail.com,**](mailto:Khan-asifnadimkhan@gmail.com) **Md. Atiar** [**Rahman-atiar@cu.ac.bd**](mailto:Rahman-atiar@cu.ac.bd)

24

25

26

# 1 List of abbreviations

2 Normal control group-NC
3 Disease control group-DC
4 Methanol extract of *Capsicum chinense*−MExCC
5 Ethyl acetate extract of *Capsicum chinense*−EAExCC
6 Petroleum ether extract of *Capsicum chinense*−PEExCC
7 Milk- ML
8 Milk with methanol extract of *Capsicum chinense*- MLMExCC
9 Sheep red blood cells – SRBC
10 Delayed-type hypersensitivity- DTH
11 Phosphate-buffered saline- PBS
12 Fetal bovine Serum – FBS
13 Hydrogen peroxide - H2O2.
14 Cyclooxygenase 2- COX 2
15 Inducible nitric oxide synthase-iNOS
16 Interleukin-6 - IL-6
17 Interleukin-1β - IL-1β
18 Tumor necrosis factor-alpha -TNF-α
19 Lipopolysaccharide – LPS
20 Nuclear factor kappa B - NF-κB
21 Mitogen-activated protein kinase- MAPK

1	**Graphical abstract**

2

3

4

5

6

7

8

9

10

11

12

13

# 1 Abstract

2 *Capsicum chinense* Jacq. organic extracts (methanol, ethyl acetate and petroleum ether) have been
3 investigated	for	their	immunomodulatory	and	DNA-damage	protecting	potential.
4 Immunomodulatory actions were undergone in SRBC-induced animal models connected with the
5 evaluation of anti-inflammatory effects in LPS-stimulated RAW264.7 macrophage cells. Fenton
6 reagent-based induction method was applied to measure the DNA damage protection effects. The
7 results displayed that higher dosage of *Capsicum chinense* methanol extract had a suppressive
8 effect on the immunological response. The extracts minimized the pro-inflammatory mediators
9 involving COX-2 and iNOS, and reduced the cytokines (TNF-α, IL-β, and IL-6) in LPS-stimulated
10 RAW264.7 macrophage cells. The petroleum ether and methanol extracts were found to exhibit a
11 more pronounced cytotoxic impact on the Hela cell line compared to the ethyl acetate extract. The
12 higher dose of the extract was found to display a negative impact, while a lower dose reversed the
13 immune reaction. The findings suggest that *C. chinense* extracts have the strong potential to
14 regulate an innate immune system in animal models.

15

16 **Keywords:** Antioxidants; *Capsicum chinense* Jacq.; DNA damage-protecting activity;
17 cytotoxicity; anti-inflammatory; immunomodulatory

18

19

20

21

22

23	**.**

# 1 1. INTRODUCTION

2 Immunomodulation refers to any alteration in the immune response, including the induction,
3 expression, amplification, or inhibition of any component or stage of the immunological response.
4 In Some people, including immunocompromised patients, immune response must be made better.
5 Whereas suppression of immune response is thought for others, such as transplant recipients,
6 patients with autoimmune diseases, allergic and inflammatory disease patients (Schijns et al.
7 2020). Apart from being specifically stimulatory or suppressive, certain agents normalize or
8 modulate pathophysiological processes and are hence called ‘immunomodulatory agents’ (Jantan,
9 Ahmad, and Bukhari 2015). The modulation of immune response with the aid of various bioactives
10 to alleviate certain diseases is an active area of interest, while immunomodulation using medicinal
11 plants scantily serve as an alternative to conventional chemotherapy for a variety of diseases,
12 especially when host defense mechanism needs to be activated under the conditions of impaired
13 immune response or when a selective immunosuppression is desired in situations like autoimmune
14 disorders.
15 Many plants and their isolated constituents have been shown to potentiate immunity and
16 exert anti-inflammatory, anti-stress? and anti-cancer effects by modulating the immune functions.
17 Nonetheless, medicinal plants and plant-derived products have yet to make significant
18 breakthrough in developing adjuvants to be used in vaccination programs or immunosuppressants
19 because of inadequate attention for plant-based immunomodulators. Fortunately, some plant
20 products are being investigated for immune response modifying activity and plethora of a plant-
21 derived materials (proteins, lectins, polysaccharides, etc.) are manifested to stimulate the immune
22 system	(Valdés-González	et	al.	2023).	Some	of	the	plants	including	*Asparagus*
23 *racemosus*, *Azadirachta indica*, *Ocimum santum, Panax ginseng*, *Polygala senega*, *Tinospora*
24 *cordifolia*, *Viscum album*, are well established for their immunomodulatory activity (Estrada et al.
1 2000; Mediratta, Sharma, and Singh 2002). However, a strong surveillance of newer sources of
2 plant-based immunomodulators is still inexorable? for prospective therapeutic approaches. Strategy
3 needs to be adopted to create innovative immunomodulators from plants and their active
4 metabolites.
5 *Capsicum chinense* Jacq. has been used medicinally for centuries to treat a wide range of
6 conditions. Several researchers have talked about how *C. chinense* Jacq. has been used in Chinese
7 medicine for a long time and what the likely scientific basis is for such uses (Bhagowati and
8 Changkija 2009). *C. chinense* Jacq. fruits have been used for a long time as a folk medicine to
9 help restore muscle tone after hard physical work Hot infusion (Carmichael 1991) of *C. chinense*
10 Jacq. is used for aches and pains in the teeth and muscles. The use of *C. chinense* as an essential
11 spice or spicy vegetable is a common use in Indian subcontinent. Bioactive capsaicinoids from *C.*
12 *chinense and* other hot chilis distinctively serve as anti-tumor, anti-cancer, antioxidant, and anti-
13 obesity molecules (Hsu and Yen 2007). Considering the pharmacological importance of *C.*
14 *chinense*, it necessitates to further investigate the immunomodulatory and relevant biological
15 functions of *C. chinense* and its products.
16 Numerous studies, both experimental and epidemiological, have investigated the potential of
17 natural compounds to modulate the immune system and reduce inflammation (Yahfoufi et al.
18 2018). The natural compounds possess the capacity to alter the expression of various pro-
19 inflammatory genes, including multiple cytokines, lipoxygenase, nitric oxide synthases, and
20 cyclooxygenase. Additionally, they exhibit antioxidant properties by protecting DNA damage
21 which aids in the regulation of inflammatory signaling (Malireddy et al. 2012; Santangelo et al.
22 2007). This study aimed to evaluate the immunomodulatory functions of *C. chinense* organic
23 extracts along with their anti-inflammatory and DNA damage protecting capacity.

# 1 2. Materials and methods

2 **2.1. Chemicals and reagents**
3 The necessary chemicals such as methanol, n-hexane, ethyl acetate, petroleum ether, tris?, boric
4 acid, agarose, ferric chloride, hydrogen peroxide, plasmid, chloroform, ascorbic acid, Na2EDTA ,
5 hematoxylin (CAS No. [517-28-2](https://www.sigmaaldrich.com/TH/en/search/517-28-2?focus=products&page=1&perpage=30&sort=relevance&term=517-28-2&type=cas_number)), xylene, and eosin were procured from Sigma-Aldrich (St Luis,
6 MO). All the chemicals used in the experiment were of analytical grade.

7

8 **2.2. Collection of *Capsicum chinense* Jacq.**
9 *Capsicum chinense* Jacq. (*C. chinense* Jacq.) green fruits were obtained from a local farmer in the
10 Reazuddin Bazar area of Chattogram (GPS 22.3365°N, 91.8303°E). Professor Dr. Shaikh
11 Bokhtear Uddin of the University of Chittagong's Department of Botany, an ethnobotanist, verified
12 the *C. chinense's* physical description and taxonomical identity. The Departmental Herbarium
13 received the voucher specimens, which are identified by accession number (ss020619-531).

14

# 15 2.3. Preparation of extracts

16 The collected *C. chinense* Jacq. samples were shade-dried and mechanically crushed into a coarse
17 powder (Sonipat, Heavy Duty10 Electric Dry Masala & Herbs Grinder, Swing Type 2300W
18 Haryana, India). The resulting powder was defatted using n-hexane and then progressively
19 extracted using petroleum ether, ethyl acetate, and lastly methanol, giving each solvent three days
20 to work. The filtrates were then dried in a rotatory evaporator (RE200, BIBBY Sterilin Ltd.
21 Staffordshire, UK) at medium pressure, and the resulting crude extracts were stored in the
22 refrigerator at 4°C until further use. The sample was henceforth designated as methanol extract of
1 *C. chinense* (MExCC); ethyl acetate extract of *C. chinense* (EAExCC), and petroleum ether extract
2 of *C. chinense* (PEExCC).

3

4 **2.4**. ***In vivo* Immunomodulating activity of *C. chinense* Jacq.**

# 5 2.4.1. Experimental design

6 Long Evans rats of both sexes weighing 90–120 g were utilized for immunological investigations
7 acquired from the International Centre for Diarrheal Disease Research, Bangladesh (icddr,b,
8 Dhaka). The animals were then randomly categorized into 7 groups, with 6 animals in each group,
9 depending on the concentration of the drug given to them. The animals were housed in cages with
10 rice husk serving as their bedding. The animals were placed in polycarbonate transparent rat cages
11 (430 mm long, 290 mm wide, and 201 mm high) maintaining at temperatures ranging from (24 ±
12 2) ºC and a dark-light cycle lasting 12 h. The animals were given access to an unlimited supply of
13 their regular pellet diet and water. The rats were acclimatized for a week. Animal handling and cares were confirmed under an ethical guideline endorsed
14 by the Institutional Ethical Committee of the Faculty of Biological Sciences of the University of
15 Chittagong (Ethical approval no. AERB-FBSCU-20230605-(1)).
16 The animals were grouped and treated with MExCC as follows:
17 Group I (NC) - Normal healthy control rats treated with water.
18 Group II (DC): Disease control rats treated with antigen.
19 Group III (ML) – Rats treated with milk.
20 Group IV (MLMExCC 100) - Rats treated with milk + 100 mg/kg bw of MExCC.
21 Group V (MLMExCC 200) - Rats treated with milk + 200 mg/kg bw of MExCC.
22 Group VI (MExCC 100) - Rats treated with 100 mg/kg bw of MExCC
1 Group VII (MExCC200) - Rats treated with 200 mg/kg bw of MExCC
2 The dose volume was calculated to be not more than 1 mL of drug preparation per animal. Control
3 animals received 1 mL of water (Bin-Hafeez et al. 2003).

4

# 5 2.4.2. Immunization

6 Blood was collected from young mature, healthy sheep from Chittagong Veterinary and Animal
7 Science University (CVASU), Chittagong. After collecting sheep red blood cells (SRBC) in
8 Alsever's solution, they were washed three times in phosphate-buffered saline (PBS), and then
9 their	concentration	was	brought	up	to	0.5	x	10^9^	cells/mL.
10 On the seventh day, an insulin syringe was used to give 0.5 x 10^9^ SRBC cells/mL intraperitoneal
11 y to all of the rats. This is the first dosage that was administered. In the same way, a booster
12 dosage was administered on the 11th day after the first one.

13

# 14 2.4.3. Body weight and lymphoid organ weight

15 After 14 days of therapy, the animals were anesthetized. Before sacrificing, their
16 body weights were measured, and their hearts were punctured with a needle using a 21-gauge
17 needle so that blood could be drawn. Several diagnostic procedures, including total hemocyte
18 count and differential count were carried out using the collected blood sample. In addition, the
19 serum was produced to analyze the serum's albumin and globulin levels. Following the collection
20 of blood, the animals were sacrificed, and the weight of organs such as the liver, thymus, and
21 spleen was noted. Lymphoid tissues, such as the spleen, the thymus, the liver, and the kidney, were
22 preserved in phosphate-buffered formalin so that histological examinations could be carried out
23 later as a continuation of this line of study.

1

# 2 2.4.4. Determination of total serum protein and albumin: globulin ratio

3 This was calculated using the Biuret technique, and the absorbance was measured using an
4 ultraviolet spectrophotometer at 540 nm.

5

# 6 2.4.5. Total leukocyte count

7 Total leukocyte was counted by WBC diluting fluid using a Hemocytometer.

8

# 9 2.4.6. Differential count of white blood cells

10 The blood smear was made on a fresh glass slide. After 3 min in methanol, the smear was dried
11 and ready to be examined. The slide was air-dried, then submerged in Field's solution B for 5 s.
12 After that, it was washed and hung up to dry. After 15 s, Field's solution A was used to stain the
13 sample. The stained slides were then washed and dried. The stained slides were examined under
14 the microscope.

15

# 16 2.4.7. Hypersensitivity reaction

17 On the final treatment day, all the animals were sensitized by injecting 0.15 ml of
18 0.025 × 10^9^ SRBC/ml subcutaneously into the subplanter region of theright hind paw using an insulin syringe. This
19 was done on the day that the treatment was finished. Then the thickness of the rats’ footpad was
20 measured using a spheromicrometer (0.01 mm pitch) after 4hs, 8hs, and 24hs.

21

# 22 2.4.8. Histopathological analyses

1 Rat spleen and thymus specimens were removed, washed with sterile saline to remove any blood
2 stains, stored in 10% buffered formalin, and then embedded in paraffin wax. The inserted tissue
3 blocks were divided into 5 µm layers using a microtome device. Hematoxylin and eosin (H&E)
4 were used to stain dewaxed sections. Using a light microscope, histopathological analyses were
5 performed.

6

# 7 2.5. Anti-Inflammatory activity against LPS-stimulated RAW264.7 macrophages

8 **2.5.1. Cell lines and culture condition**
9 RAW264.7 macrophage cells were acquired from the ATCC (American Type Culture Collection)
10 and grown in Dulbecco's modified Eagle's medium (DMEM), which included 10% (v/v) fetal
11 bovine serum (FBS) and 1% (v/v) P/S at 37°C in a humidified environment containing 5% carbon
12 dioxide.

13

# 14 2.5.2. Morphological Study Using an Inverted Light Microscope

15 The morphological investigation was carried out according toFair and Tor
16 2014. Briefly, 10^5^ cells were plated into each well of a six-well plate, and then the plate was
17 placed in an incubator for 24 h to allow the cells to connect. After that, the medium was withdrawn,
18 and a new medium containing treated samples at their IC50 concentration was added. The cells,
19 both treated and untreated, were then incubated once more for a total of 72 h. Under an inverted
20 light microscope, morphological changes in the cells were noticed after they had been subjected
21 to incubation (Olympus, Tokyo, Japan).

22

# 23 2.5.3. RT-qPCR for analysis of gene expression

1 Using TRIzol reagent (Invitrogen), total RNA was extracted from both controls (DMSO alone)
2 and treated (with 1 μg of LPS) RAW 264.7 macrophages, and the amount of RNA extracted was
3 determined using a NanoDrop spectrophotometer. Purified total RNA (around 1 μg) was reverse-
4 transcribed into cDNA in a final volume of 10 μL using a High-capacity RNA to cDNA Master
5 Mix kit (Applied Biosystem), as per the manufacturer's instructions. Using the freshly synthesized
6 cDNA as a template, RT-qPCR was conducted using SYBR qPCR mix (Nippon Gene) according
7 to a standard technique (Haque et al. 2020). Primer sequences (Table 1) used to quantify genes
8 involved in inflammation are listed. The ΔCt values were calculated and the standard deviation of
9 the Ct values of the ΔCt values was also calculated, which were used to determine the ΔΔCt values.
10 And then, all data was collected and calculated as 2 ^-^△△Ct.

11

# 12 2.6. DNA damage protecting activity

13 **2.6.1. Preparation of Reagents**
14 Reagents were prepared according to the description by Thangara (Thangaraj 2019). In order to
15 create a 1 L solution of 10X TBE stock, the following steps should be followed: The procedure
16 involves the dissolution of 108 g of Tris and 55 grams of boric acid in 800 mL of distilled water.
17 Incorporate 40 mL of a solution containing 0.5 M of Na2EDTA with a pH value of 8.0. Set the
18 volume to a value of 1L. To prepare a solution of 0.5X TBE running buffer with a volume of 1 L,
19 mix 50 mL of a 10-fold concentrated Tris-Borate-EDTA (TBE) stock solution with 950 mL of
20 sterile water, ensuring thorough mixing.

21

# 22 2.6.2. Agarose gel

1 To begin, put the required amount of 0.4 g agarose (0.8 percent gel) was taken in a conical flask by
2 weighing it. The agarose was then heated to dissolve it in 50 mL of 1x TBE running buffer. Give
3 everything a brisk swirl now and again to keep it evenly combined. To better combine the two
4 components, ethidium bromide (at a concentration of 0.5 g/mL) was added to the molten gel a few
5 minutes later when the temperature had dropped to around 60°C. In the mold, insert a comb that
6 is the appropriate size and form. Pour the gel into the gel mold after allowing it to cool. The gel
7 could solidify and be used for loading samples when the comb was taken out and the gel box was
8 set up.

9

# 10 2.7. DNA damage protecting assay

11 Fenton's reagent was prepared by adding 0.65 g of ferric chloride (FeCl3) (80 mM) to a mixture of
12 0.45 g of ascorbic acid (50 mM) and distilled water. Finally, 1.5 μL of 30 mM hydrogen peroxide
13 (H2O2) was added, bringing the total volume of the solution to 50 mL after vigorous shaking (Lee
14 et al. 2002) was slightly altered to assess the DNA-protecting potential of plant samples. Extracts
15 of varying quantities were tested for their ability to quench the hydroxyl radical generated by
16 Fenton's reagent using the plasmid pBR322. Thereafter, 3 μL of plasmid DNA was added to 5 μL
17 of Fenton's reagent (30 mM H2O2, 50 mM ascorbic acid, and 80 mM FeCl3), and then varying
18 volumes of extracts (0, 20, 40, 60, 80, 100 μg/mL, and final volume of the reaction mixture was
19 made 20 μL by adding nuclease-free water) to each PCR tube. One test tube containing plasmid
20 and Fenton's reagent, but no extracts was kept as a negative control. When compared to the
21 negative control of extract plus Fenton's reagent, the positive control was plasmid alone. After that
22 the reaction mixture be allowed to ?at 37°C for half an h. Each test tube was then diluted with
23 bromophenol blue dye (0.25% in 50% glycerol) after incubation. Thereafter, a precast agarose gel
1 (0.8%) was loaded with all the tubes and placed in electrophoresis equipment for DNA fragment
2 separation at 90 V for 1 h using ethidium bromide labeling. Running buffer was a 1x TBE solution
3 that was put into the tank and used to submerge the gel in the buffer. DNA bands were finally seen
4 in the gel. Sample and plasmid alone resulted in distinct bands. UV microscopy of DNA stained
5 with ethidium bromide (EtBr) revealed bands of DNA indicative of fragments formed from radical
6 damage after being exposed to hydrogen peroxide (H2O2). When samples were introduced to the
7 plasmid and Fenton's reagent, the antioxidant reduced the damage of hydroxyl radicals in a dose-
8 dependent manner. The BIO-VISION V17.06 Gel Documentation system was used to examine the
9 bands.

10

11 **2.8. Cytotoxic effect of *C. chinense* Jacq.**
12 Briefly, DMEM (Dulbecco's Modified Eagles' medium) containing 1% penicillin-streptomycin
13 (1:1) and 0.2% gentamycin and 10% fetal bovine serum (FBS) was used to maintain the cell lines
14 BHK-21, a baby hamster kidney fibroblast cell line, HeLa, a human cervical carcinoma cell line,
15 and Vero, an African green monkey kidney epithelial cell line. BHK-21 Cells (3×10^4^/200 µL),
16 HeLa Cells (4.0×10^4^/200 µL), and Vero cells (3×10^4^/200 µL) were seeded onto a 48-well plate
17 and incubated at 37°C+5% CO2. The next day, 50 µL of the sample (filtered) was added to each
18 well. Cytotoxicity was examined under an inverted light microscope after 48h of incubation.
19 Duplicate wells were used for each sample**.**

20

# 21 2.9. Statistical analysis

22 All information is shown as the mean ± SD of 6 animals. The data were assessed using the t-test,
23 one-way ANOVA, two-way ANOVA, and Tukey’s multiple range post hoc tests in the statistical
1 program Graph-Pad Prism v8.4.3. The results were deemed to be substantially different at the
2 levels of *P ≤ 0.05, **P ≤ 0.01, and ***P ≤ 0.001.

3

4 **3. RESULTS**
5 **3.1. Immunomodulatory Activities**
6 **3.1.1. Effect of *C. chinense* Jacq. extracts on body and organ weight**
7 The effects of *C. chinense* on the body and organ weights are summarized in Tables 2 & 3 All
8 groups, except the highest extract dosage group, gained considerably more weight compared to the
9 NC group. The final body weight of milk-treated group was somewhat higher compared to the NC
10 group. However, the body weight of both MLMExCC100 and MLMExCC200 groups were quite
11 similar. The MExCC100 group had a higher final body weight than the other groups. The highest
12 dose was found to have minimal effect on the body weights than the other doses.
13 There was no statistically significant difference in the total weight of the livers among the groups.
14 The same is true for the spleen, which shows no significant differences between the treatment
15 groups and the normal control group. The thymus weight achieved by the highest dose group was
16 found to be significantly (P < 0.05) different from that of other groups while other doses had no
17 significant differences (Table 3).

18

# 19 3.1.2. Delayed type hypersensitivity test

20 Figure 1 presented the effects of *C. chinense* extracts on the delayed type of hypersensitivity that
21 was noticed for every group of SRBC-induced animals. A remarkable increase in volumes was
22 recorded for both doses. After 4 h of SRBC injection, the thickness of paw was recorded 19.8 ±
23 3.89%, 20.2 ± 4.76 %, 34.0 ± 6.52 %, 34.0 ± 5.48%, 34.0 ± 4.18%, 58.8 ± 18.59%, and 35.6 ±5.85
1 % respectively for NC, DC, ML, MLMExCC100, MLMExCC200, MExCC100, and MExCC200.
2 After 24 h the thickness of paw was recorded 0.8 ± 1.09 %, 18.8 ± 3.56 %, 0.8 ± 1.09 %, 2.40 ±
3 1.81 %,.2.40 ± 2.88%, 5.40 ± 2.50%, 32.4 ± 4.50% for NC, DC, ML, MLMExCC100,
4 MLMExCC200, MExCC100, and MExCC200. However, the effects of ML, MLMExCC100,
5 MLMExCC200, MExCC100, and MExCC200 are not significant compared to the NC group
6 (Figure 1).

# 7 3.1.3. Hematological assays

8 The total leukocyte counts and differential leukocyte counts are presented in Figures 2 (A & B)
9 respectively. When compared to the NC group, total leukocyte count was observed to be elevated
10 in the MExCC100 as well as in the ML, MLMExCC100, and MLMExCC200. However, an
11 increase in the extract concentration has resulted in a further reduction in the overall number of
12 leukocytes. The percentage of neutrophils was found to be maximum in the MLMExCC200 group
13 and also in other extracts. The monocyte and eosinophil count for treatment groups were not
14 changed significantly compared to the NC group.

15

# 16 3.1.4. Serological parameter

17 The serological parameters displayed in Figure 3, showed that the blood protein level for the
18 extract at MExCC100 was found to be significantly higher than that of the other extracts. The
19 protein level was recorded at 5.74 ± 0.19, 5.36 ± 0.11, 5.74 ± 0.26, 6.02 ± 0.16, 6.18 ± 0.18, and
20 6.22 ± 0.20 gm/dL for NC, DC, ML, MLMExCC100, MLMExCC200, and MExCC100
21 respectively. On the other hand, in the case of MExCC200, the quantity of serum protein (5.46 ±
22 0.19 gm/dL) was decreased. Figure 3 also summarizes the albumin/globulin ratio for different
23 treatments. The ratio was increased in all extracts; however, the highest dose was noticed to
1 slightly reduce the ratio. Above all, the AG ratio for the extracts was insignificant compared to
2 NC.

# 3 3.1.5. Histopathological assay of spleen and thymus

4 Histopathological examination of the normal control group's spleen revealed no abnormalities. The
5 white pulp of the spleens of rats in the control group had clearly defined T cell regions that
6 surrounded the central arteries and formed periarteriolar lymphoid sheaths (PALS). In addition to
7 this, the follicles contain germinal centers that are actively undergoing mitosis. At the follicular
8 edge, there was a mantle zone, and beyond that, there was an enormous marginal zone. In Figure
9 4A, the marginal sinus was also easily seen, and the white and red pulp in between the border was
10 shown in a manner that was obvious and clear. In Figure 4B, the effects of SRBC can be seen as a
11 dramatic reduction in the volume density of the lymph follicles and a large rise in the amount of
12 red pulp. Additionally, the medulla includes more developed lymphocytes, conspicuous epithelial
13 cells, admixed macrophages, Hassall's corpuscles, and dendritic cells. Although the disease control
14 group's spleen exhibited an absence of full germinal centers in the white pulp of the spleen tissue,
15 these centers were represented infrequently, had smaller diameters and were accompanied by a
16 reduction in lymphocyte count. Many follicles contain germinal centers, and the boundary between
17 the white pulp and the red pulp was well delineated in the ML-treated group's representation in
18 Figure 4C. The spleen of the groups of rats to whom the MLMExCC100, MLMExCC200, and
19 MExCC100 were given showed moderate white pulp with an increase in the number of
20 reticuloendothelial cells in the red pulp (Figures 4D, 4E, 4F). The administration of MExCC200
21 led to a discernible decrease of the volume density of the lymph follicles as seen in Figure 4G as
22 well as a discernibly greater expansion of the red pulp and exhibited a lack of full germinal centers
1 in the white pulp in all the samples, those germinal centers that were present showed up
2 infrequently, had reduced dimensions, and was accompanied by a loss of lymphocytes.
3 The NC group in Figure 5A has a thymus that is made of a darkly colored cortex that has a high
4 packing of tiny immature lymphocytes. The medulla was seen to have a lighter coloring and less
5 densely cellular composition than the cortex. The thymus demonstrated a decline in the cellular
6 density of cortical lymphocytes, as well as worsening alterations in the remaining epithelial and
7 stromal cells, as evidenced by cytoplasmic vacuolization in Figure 5B, which depicts the DC
8 group. A seemingly typical structure was shown by the thymus in Figure 5C, which included
9 highly populated areas of growing T cells in the thymic cortex and a lesser number of epithelial
10 cells associated with these T cells. In the milk-treated group, the medulla commonly had mature T
11 cells that were bigger in size. Additionally, the medulla contained a greater abundance of epithelial
12 cells and other cell types. The groups of rats to whom the MLMExCC100, MLMExCC200, and
13 MExCC100 were given, the thymus showed a moderate increase in the number of lymphocyte
14 population in both the cortex and the medulla, and macrophages phagocytized apoptotic debris
15 (Figures 5D, 5E, 5F). Treated with MExCC200 groups exhibited a significant decrease in the
16 number of trabeculae smooth muscle cells and a predominance of collagen fibers.

17

# 18 3.2. Anti-inflammatory activity

19 **3.2.1. Anti-inflammatory effects of *C. chinense* Jacq. in RAW 264.7 cells**
20 The anti-inflammatory effects of *C. chinense* extracts are presented in Figure 6. The protein
21 expression of both iNOS and COX-2 was found to be inhibited by the *C. chinense* Jacq. extracts.
22 The LPS-pretreated group had significantly higher levels of iNOS and COX-2 expression than the
23 non-LPS group. However, *C. chinense* extract treatment demonstrated the suppression of iNOS
1 and COX-2 expression. These findings demonstrate that the plant material has powerful inhibitory
2 effects on the production of NO and early inflammatory factors (Figure 6) and that NO production
3 can be inhibited by inhibiting the expression of iNOS. Additionally, these findings demonstrate
4 that the plant material inhibits the production of early inflammatory factors.

5

# 6 3.2.2. Inhibitory effects of *C. chinense* Jacq. on pro-inflammatory cytokine production

7 The inflammatory cytokines TNF-α, IL-6, and IL-1β are all secreted by macrophages after being
8 stimulated by LPS. The possibility that the extracts would have an inhibitory impact on the
9 production of cytokines was investigated (Figure 6). In LPS-stimulated RAW 264.7 cells, the
10 expression of IL-6, and IL-1β was dramatically suppressed in the *C. chinense* Jacq treated group.
11 And the expression of TNF-α was also suppressed. Additionally, these findings demonstrate that
12 the plant material inhibits the production of early inflammatory factors.

13

# 14 3.3. Effect of *C. chinense* extracts on DNA damage protection

15 When run through an agarose gel electrophoresis (lane 1), the DNA that was extracted from the
16 pBR322 DNA plasmid appeared as two distinct bands: the band that moved more quickly
17 corresponded to the native form of supercoiled circular DNA (sc DNA), while the band that moved
18 more slowly was the open circular form of DNA (R- Form). After Fenton's reaction of DNA in the
19 presence of H2O2, the DNA was cleaved to linear form (L-DNA), which shows that the OH radicals
20 produced by H2O2 induced DNA strand scission. DNA was cleaved to linear form after Fenton's
21 reaction of DNA in the presence of H2O2. When compared to the untreated lane-1, the production
22 of DNA in a repairing form was promoted by adding petroleum ether extract to the reaction mixture
23 at varied concentrations (100, 80, 60, 40, and 20 μg/mL) in lanes 3−7. This resulted in a
1 concentration-dependent action of hydroxyl radical scavenging, which led to the recovery of some
2 of the scDNA. In contrast, there is not even a shred of DNA present in lane 2 of the experiment
3 (Figure 7).
4 The protection of DNA from damage was carried out by the EAExCC mediated via Fenton's
5 reagent. The electrophoresis gel pattern showed that the varying concentrations of extract (100,
6 80, 60, 40, and 20 μg/mL) produced distinct patterns (Figure 7). When plasmid DNA was treated
7 in Fenton's reagent without an extract, the reaction produced strand breaking that led to the relaxed
8 form (R- form) (Lane 1−2). However, there was no evidence of recovered native DNA. When
9 incubated with EAExCC, converting native supercoiled DNA did not demonstrate a dose-
10 dependent way nor did it exhibit any dose-dependent effects (Lane 3−7).
11 In comparison to the untreated lane-1, the formation of L-DNA in a repairing form was stimulated
12 by adding MExCC to the reaction mixture at varying concentrations (100, 80, 60, 40, and 20
13 μg/mL) in lanes 3−7. This led to partial scDNA recovery due to a concentration-dependent
14 hydroxyl radical scavenging effect. In comparison, there is not even a trace of DNA in lane 2
15 (Figure 7).

16

# 17 3.4. Cytotoxic effects of *C. chinense* Jacq. extracts on different cell lines

18 Extracts of *C. chinense* Jacq. were tested on Vero, BHK-21, and HeLa cell lines to determine their
19 cytotoxic activity. In the presence of PEExCC, EAExCC and MExCC, more than 95% of BHK-
20 21 cells were able to survive (Figure 8A). On the other hand, the survival rate of Vero cells that
21 were treated with PEExCC was < 5%, while the survival rate of same cells treated with EAExCC
22 and MExCC was greater than 95% (Figure 8A). On the HeLa cell line, EAExCC had no discernible
1 effect. Using these extracts, > 95% of the cells were able to survive. However, the survival rate
2 was less than 5% in samples taken from PEExCC and MExCC (Figure 8B).

3

# 4 4. Discussion

5 Natural immunotherapies are regarded to be promising alternatives to chemotherapies and
6 vaccines because of their broad range of action, environmentall friendly approaches, and cost
7 effectiveness (Gupta et al. 2022). After 15 days of consecutive food supplementation in Long Evans rats,
8 the effects of the extracts on the potentiation of the immune system were explored in this research.
9 the immunomodulatory activity of *C. chinense* Jacq. combined with milk was examined using the
10 long Evan rat model. This is the first time that this information has been reported. During our
11 research, the extracts had an impact on both the overall body weight of the rats as well as the relative weight
12 of their important organs like the liver. Despite this, there was not a discernible difference between
13 the treatment group and the control group in terms of the bw of the animals. However, substantial
14 changes were seen between the dosages of 100 and 200 mg/kg bw in the groups that were treated.
15 The body weight of the groups that got the highest dosage of MExCC200 of tested extracts changed
16 less than the body weight of the groups that acquired the lowest dose of MExCC100 of extracts of
17 the experiment that lasted for 14 days. The bw continuously decreased as the extract dosage was
18 raised, which suggests that at dosages over 200 mg/kg bw, the substance is growth-inhibiting rather
19 than growth-enhancing. This is because growth-inhibiting doses are higher than growth-enhancing
20 ones.
21 The findings are supported by evidence gleaned from the liver, which is a vital organ that responds
22 quickly to any drug. The greatest dosage, which was 200 mg/kg, resulted in a greater liver mass
23 compared to the lowest dose, which was 100 mg/kg. This indicates that it is hyperactive to offset
1 the impact of the extract. But in the case of other extracts the liver weights were normal, this
2 suggests that the natural activity of the liver was preserved by the other extracts. There was no
3 difference in the weight of the spleen between the monitoring group and the recovery group, but
4 there was a significant difference in the weight of the thymus between the other extract groups and
5 the high-dose extract group. The researchers Bin-Hafeez et al. (Bin-Hafeez et al. 2003) and
6 Sumalatha et al. (Bhat, Ballal, and Acharya 2012) evaluated the immunomodulatory effectiveness
7 of fenugreek and Salacia chinensis extracts in mice. They found that there was no substantial
8 change in the animals' bw, and there was also no influence on the size of the spleen.
9 The animal's hematological characteristics show and decide one of the initial immune responses
10 to a pathogen. The total leukocyte counts as well as the differential leukocyte count were assessed,
11 as was the control group as well as the pretreatment groups with varying extract dosages. In this
12 investigation, we investigated the immunological components and mediators that were present in
13 rats that had been given extracts of *C. chinense* Jacq. that had been subjected to a challenge. After
14 being forcibly fed on each of the several extracts, the rats' hematologic parameters were found to
15 have undergone a considerable shift, as shown by the findings of our study. At dosages of ML,
16 MLMExCC100 of the *C. chinense* Jacq. extracts, there was a considerable rise in the WBCs.
17 MLMExCC200, MExCC100 extract exhibited a considerable rise in the WBCs compared to the
18 untreated and negative control groups with a modest increase from the positive control group. On
19 the other hand, the WBCs count declined in the highest dose feeding extract group.
20 Also, neutrophils are part of the cell-mediated immune responses that are responsible for the innate
21 immunity that contributes to the clearance of foreign bodies by recognizing and migrating toward
22 the foreign body, engaging in phagocytosis, and destroying the foreign agent (Jr et al. 2001). The
23 presence of a wide variety of chemicals, macronutrients, and micronutrients in herbs may be
1 responsible for the higher percentage of neutrophil adhesion (Nfambi et al. 2015). This makes a
2 significant contribution to the capacity of neutrophils to migrate in the direction of an infectious
3 pathogen. Because of their crucial function in calcium and bone metabolism, micronutrients like
4 vitamin D are required for the formation of polymorphonuclear neutrophils. This is because these
5 micronutrients help maintain healthy bone tissue. In the current study, the groups ML,
6 MLMExCC200, MLMExCC100, and MExCC100, had the maximum neutrophil count of 59.2%,
7 59.2%, 60.8%, 60% WBC/mm^3^ indicating the blood cells were being triggered to launch a potent
8 immune response. The result showed that the other extracts elicit a stronger immune response than
9 MExCC200. Since the plant extract increases the phagocytic activity of the neutrophils, it might
10 be employed to boost the cell-mediated immune response in cases when the immune system is
11 already impaired.
12 According to the categorization of hypersensitivity responses developed by Coombs and Gell in
13 1975 (Rajan 2003), the DTH reaction is a cell-mediated immune response of the type IV kind. The
14 increase in footpad thickness of the rats that were subjected to *C. chinense* extracts in this study
15 could be attributed to the ability of the extract to activate lymphocytes and their accessory cell
16 types, leading to an enhancement in the production of antibodies in animals that had previously
17 been immunosuppressed, thereby increasing cell-mediated immunity. The rats were given *C.*
18 *chinense* Jacq. extracts via intraperitoneal injection. Vitamins A and C have been identified as
19 being present in *C. chinense* Jacq.(Kantar et al. 2016). These substances activate the immune
20 system by promoting T-cell proliferation, increasing cytokine release, and synthesis of
21 immunoglobulins (Mora, Iwata, and Von Andrian 2008). All of these factors play an essential role
22 in the inflammatory response, which was seen as an increase in the thickness of the foot pad. In
23 addition, the extract of *C. chinense* Jacq. contains amino acids that play a crucial role in the
1 production of immunoglobulins and major histocompatibility complexes, both of which are
2 required for the successful mediation of the DTH response. Trace elements are necessary for the
3 proliferation of T-cells and Langerhan cells as well as the activity of lytic enzymes, both of which
4 are crucial components of the DTH response to antigen. Trace elements may be found in a variety
5 of plant and animal foods. The delayed type of hypersensitivity reaction is one of the parameters
6 that is often used to evaluate an animal's cell-mediated immune response. Evaluation of the
7 immunomodulatory activities of *Momordica charantia ghrita* extract carried out on albino rats
8 was carried out by Prasad et al. (Prasad, Jain, and Dorle 2010) using a delayed type hypersensitivity
9 experiment. When the medicine was given at a dosage of 350 mg / kg per day, the scientists found
10 that there was a significant increase in the volume of the paws. In the instance of the rats that were
11 given herbal medications, Pradhan et al. (Pradhan, Panda, and Tripathy 2009) demonstrated that
12 there was an enhanced hypersensitive response. In the same vein as earlier findings, Bin-Hafeez
13 et al. (Bin-Hafeez et al. 2003) demonstrated that when fenugreek was utilized at a concentration
14 of 50 mg/kg, a significant increase in delayed type hypersensitivity response was observed in
15 comparison to the control. This was the case when looking at the results of the experiment. In
16 addition, Fulzel et al. (Fulzele et al. 2002) demonstrated that *Ashtamangala ghrita* has the ability
17 to shed light on an elevated delayed type hypersensitivity reaction. In line with the findings
18 described in the previous papers, our high-dose extracts induced a more delayed type
19 hypersensitivity reaction. Since there was a dose-dependent increase in paw size in response to
20 antigen, the findings of the research demonstrated that the MExCC may be utilized to stimulate
21 the immune system.
22 The ratio of an individual's serum protein to their serum albumin globulin is one of the early
23 markers that an individual's blood chemistry is normal. If there was a shift in the serum protein
1 content as well as the albumin ratio, this would provide us with a clue about the individual's
2 changed immune response state. According to the findings of the current study, the serum protein
3 level and albumin globulin ratio are found to be lower in the case of higher concentrations of
4 MExCC200 of the drug. However, in the other concentrations of extracts test, the group showed
5 an increase in serum protein and albumin ratio, indicating that higher immune responses might
6 have contributed to the serum protein in terms of a variety of different molecules such as
7 immunoglobulins and other humoral factors.
8 Several	anti-inflammatory	bioactivities	in	plants	have	recently	been	linked	through
9 epidemiological research. As a result, conventional pharmaceuticals or natural products may be a
10 source of new anti-inflammatory active molecules, which may eventually lead to novel therapies
11 with less hazardous side effects (Yuan et al. 2016; Beg et al. 2011) . By preventing cell damage
12 and death from inflammation and apoptosis, antioxidants play a crucial role in redox pathways
13 (Lin et al. 2016). Over activation of macrophages and subsequent excessive inflammation may
14 result from oxidative stress brought on by LPS or other triggers (Xu et al. 2015). Here, we provided
15 strong evidence that the extracts inhibited LPS-induced generation of TNF-α, intracellular ROS in
16 RAW 264.7 macrophages. This provides evidence that the extracts have anti-inflammatory
17 qualities that may help with a variety of inflammatory symptoms. Inhibition of NF-kB and MAPK
18 pathway activation in LPS-stimulated RAW 264.7 cells have been shown before to reduce iNOS,
19 COX-2, TNF-α, and IL-6 production (Huang et al. 2015). We found evidence that the extracts had
20 potent antioxidant effects, maybe because of the large quantities of polyphenolic chemicals they
21 contain. Based on the results of a previous research (Zuo et al. 2019), it was determined that LPS
22 induces oxidative stress, which in turn may activate macrophages and result in an inflammatory
23 response that is too strong. So, we dug further into the antioxidant actions that lay behind the
1 reduction in LPS-induced macrophage activation. In macrophages, the production of ROS is
2 quickly boosted in response to LPS stimulation. Furthermore, this reaction induces DNA damage
3 and results in an increase in inflammatory cytokines and enzymes (Zhang et al. 2003). During
4 inflammation, the immune system produces cytokines (TNF-α, IL-6, IL-1β) and enzymes (iNOS,
5 COX-2) that promote further inflammation. To our surprise, our extract suppressed the production
6 of these cytokines and enzymes. Local inflammation and sepsis are primarily mediated by
7 interleukin-6 and tumor necrosis factor alpha (Leon, White, and Kluger 1998). Increased IL-1β
8 expression was seen in several human malignancies, including melanoma, colon, breast, and lung
9 tumors (Baker, Houston, and Brint 2019). This indicates that the extracts not only dampen the
10 inflammatory response, but also stimulate the production of anti-inflammatory molecules. It is
11 imperative that the precise mechanism(s) responsible for *C. chinense* Jacq. anti-inflammatory
12 properties should be investigated to target inflammatory illnesses.
13 It is well known that OH-free radicals cause damage to cellular DNA. The majority of the oxidative
14 damage that occurs to proteins and DNA is caused by OH radicals, which are produced when H2O2
15 is photolyzed by UV light. According to Guha et al. (Guha et al. 2011) , when OH radicals attach
16 to DNA, this results in strand breaking and opening, as well as fragmentation of deoxyribose sugar
17 and alteration of nitrogenous bases. In most cases, OH radicals are to blame for the oxidative
18 damage that is done to DNA, which is one of the most critical processes in the beginning stages of
19 the development of cancer (Reddy, Urooj, and Kumar 2005). All the extracts were tested to see
20 whether or not they could preserve a model DNA called pBR322 from oxidative damage. It is well
21 established that the hydroxyl radicals produced by Fenton's reaction may damage DNA, as shown
22 by the absence of the DNA band in lanes 2 of Fig 10. Even though all the extracts, with the
23 exception of EXExCC, effectively mitigated the oxidative stress and protected the DNA from the
1 hydroxyl radicals generated by Fenton's reaction, as confirmed by the presence of DNA bands (Fig
2 10) appears to be comparably most effective in maintaining the DNA's intact state respectively.
3 DNA strands may be broken and damaged by free radicals, which can ultimately lead to
4 carcinogenesis, mutagenesis, and cytotoxicity. Free radicals are notorious for their harmful effects
5 (Poorna, Resmi, and Soniya 2013; M. Kumar, Kumar, and Kaur 2011). Several groups of
6 researchers have come to the same conclusions and employed plant extracts and fractions to protect
7 DNA from oxidative damage (V. Kumar et al. 2013). In this study, the above two extracts
8 displayed considerable protective activity against free radical-mediated DNA damage and could,
9 therefore, be used in cancer prevention.
10 Furthermore, the BHK-21 and Vero cell lines were not too affected by the EAExCC and MExCC
11 showing that they are sustainable. PEExCC, on the other hand, was harmful to Vero cell lines.
12 PEExCC, and MExCC were shown to reduce the growth of the cancer cell line HeLa. The effects
13 of *C. chinense* Jacq. extracts on BHK21, vero, and cervical cancer cell lines are compared for the
14 first time in this work. Three extracts of *C. chinense* Jacq. were tested for their cytotoxic effects
15 on the HeLa cell line in the present investigation. The HeLa cell line was found to be sensitive to
16 PEExCC and MExCC cytotoxic effects. Both extracts were more effective against the HeLa cell
17 line than the EAExCC. When tested on three different cell lines, methanol extract showed the most
18 promising. Plant phytochemicals (Pan, Ghai, and Ho 2008) trigger apoptosis through the intrinsic
19 route by inducing the release of cytochrome-c in certain tumor cell types. Tomatoes, which contain
20 the carotenoid phytochemical lycopene (Hantz, Young, and Martin 2005), chilli peppers, which
21 contain the phenolic compound capsaicin (Macho et al. 1999), and celery, which contain the
22 flavonoids luteolin and curcumin (Apostolidis, Kwon, and Shetty 2007), they depolarize
23 mitochondria and induce apoptosis in human tumor cells (Cheng et al. 2005).

1

# 2 5. Conclusion

3 When compared to the capsaicin levels found in the fruits of other chilli species, it has been shown
4 that the levels of capsaicin found in *C. chinense* Jacq. fruits are very high. The properties of *C.*
5 *chinense* Jacq. that make it useful in pharmacology include analgesic, antiarthritic, anticancer, and
6 antioxidant effects. Only just a handful of research papers on *C. chinense* Jacq. have been
7 published up to this point, even though it has the potential to be a source of antioxidants in addition
8 to possessing other pharmacological properties. According to the findings of this study, the extracts

9	may safeguard DNA from the damaging effects of oxidative stress. The outcomes of this

10 investigation showed, that the immune response is greatly improved when the methanolic
11 extracts are given as a treatment. According to the results, drug extracts taken in lower amounts

12	may activate both a humoral and cellular immune response in the body. It has been shown that

13 higher dosages or doses that are greater overall, hinder the immune system's capacity to mount a
14 response. In conclusion, the extracts improve the body's immune system, however it is important
15 to note that a high concentration of the extracts may have a biochemical impact on the body.

16

# 17 Acknowledgment

18 The authors wish to acknowledge the members of the Laboratory of alternative Medicine and
19 Natural Products Research for their undeniable support to accomplish the research.

20

# 21 Authors contribution

22 **Srabonti Saha−** Resources, Methodology, data curation, data analysis, original draft writing; **Md.**
23 **Altaf Hossain −** Methodology, Data curation, investigation**; Humayra Ferdousi −**Data analysis,
1 data validation**; Jobaier Ibne Deen −**Methodology, Software, Data curation, original draft-
2 writing; **Akhlak Chowdhyry −**Resources, Data curation, Investigation; **Md. Mannan −**Data
3 curation, visualization, investigation**, Md. Asif Nadim Khan −**Supervision, validation, Data
4 curation**.** All authors have gone through the manuscript and agreed to submit it to the **Plos One**.

5

# 6 Conflict of interest

7 Authors declare that there is no conflict of interest.

# 8 Funding

9 NA

10

# 11 Data availability

12 Data will be available on request.

13

14

15

16

17

18

19

20

21

22

23

# 1 References

2 Apostolidis, E, Y-I Kwon, and K Shetty. 2007. “Inhibitory Potential of Herb, Fruit, and Fungal-
3 Enriched Cheese against Key Enzymes Linked to Type 2 Diabetes and Hypertension.”
4 *Innovative Food Science & Emerging Technologies* 8 (1): 46–54.
5 Baker, Kevin J, Aileen Houston, and Elizabeth Brint. 2019. “IL-1 Family Members in Cancer;
6 Two Sides to Every Story.” *Frontiers in Immunology* 10: 1197.
7 Beg, Sarwar, Suryakanta Swain, Hameed Hasan, M Abul Barkat, and Md Sarfaraz Hussain.
8 2011. “Systematic Review of Herbals as Potential Anti-Inflammatory Agents: Recent
9 Advances, Current Clinical Status and Future Perspectives.” *Pharmacognosy Reviews* 5
10 (10): 120.
11 Bhagowati, Raktim Ranjan, and Sapu Changkija. 2009. “Genetic Variability and Traditional
12 Practices in Naga King Chili Landraces of Nagaland.” *Asian Agri-History* 13 (3): 171–
13 80.
14 Bhat, Rama, Shwetha R Ballal, and Sadananda Acharya. 2012. “Studies on Immunomodulatory
15 Effects of Salacia Chinensis L. on Albino Rats.” *Journal of Applied Pharmaceutical*
16 *Science* 2 (9): 98–107.
17 Bin-Hafeez, Bilal, Rizwanul Haque, Suhel Parvez, Suwarna Pandey, Iqbal Sayeed, and S
18 Raisuddin. 2003. “Immunomodulatory Effects of Fenugreek (Trigonella Foenum
19 Graecum L.) Extract in Mice.” *International Immunopharmacology* 3 (2): 257–65.
20 Carmichael, J Kevin. 1991. “Treatment of Herpes Zoster and Postherpetic Neuralgia.” *American*
21 *Family Physician* 44 (1): 203–10.
22 Cheng, An-Chin, Tzou-Chi Huang, Ching-Shu Lai, and Min-Hsiung Pan. 2005. “Induction of
23 Apoptosis by Luteolin through Cleavage of Bcl-2 Family in Human Leukemia HL-60
24 Cells.” *European Journal of Pharmacology* 509 (1): 1–10.
25 Estrada, Alberto, Georgios S Katselis, Bernard Laarveld, and Branka Barl. 2000. “Isolation and
26 Evaluation of Immunological Adjuvant Activities of Saponins from Polygala Senega L.”
27 *Comparative Immunology, Microbiology and Infectious Diseases* 23 (1): 27–43.
28 Fair, Richard J, and Yitzhak Tor. 2014. “Antibiotics and Bacterial Resistance in the 21st
29 Century.” *Perspectives in Medicinal Chemistry* 6: PMC-S14459.
30 Fulzele, SV, PM Bhurchandi, VM Kanoje, SB Joshi, and AK Dorle. 2002. “Immunostimulant
31 Activity of Ashtamangal Ghrita in Rats.” *Indian Journal of Pharmacology* 34 (3): 194–
32 97.
33 Guha, Gunjan, Venkatadri Rajkumar, Lazar Mathew, and RANGASAMY ASHOK KUMAR.
34 2011. “The Antioxidant and DNA Protection Potential of Indian Tribal Medicinal
35 Plants.” *Turkish Journal of Biology* 35 (2): 233–42.
36 Gupta, Sneh Lata, Srijani Basu, Vijay Soni, and Rishi K Jaiswal. 2022. “Immunotherapy: An
37 Alternative Promising Therapeutic Approach against Cancers.” *Molecular Biology*
38 *Reports* 49 (10): 9903–13.
39 Hantz, Holly L, Leeanne F Young, and Keith R Martin. 2005. “Physiologically Attainable
40 Concentrations of Lycopene Induce Mitochondrial Apoptosis in LNCaP Human Prostate
41 Cancer Cells.” *Experimental Biology and Medicine* 230 (3): 171–79.
42 Haque, Md Anwarul, A. S. M. Ali Reza, Mst Samima Nasrin, and Md Atiar Rahman. 2020.
43 “Pleurotus Highking Mushrooms Potentiate Antiproliferative and Antimigratory Activity
44 against Triple-Negative Breast Cancer Cells by Suppressing Akt Signaling.” *Integrative*
45 *Cancer Therapies* 19: 1534735420969809. https://doi.org/10.1177/1534735420969809.
   1 Hsu, Chin-Lin, and Gow-Chin Yen. 2007. “Effects of Capsaicin on Induction of Apoptosis and
   2 Inhibition of Adipogenesis in 3T3-L1 Cells.” *Journal of Agricultural and Food*
   3 *Chemistry* 55 (5): 1730–36.
   4 Huang, Bee-Piao, Chun-Hsiang Lin, Han-Min Chen, Jiun-Tsai Lin, Yi-Fang Cheng, and Shao-
   5 Hsuan Kao. 2015. “AMPK Activation Inhibits Expression of Proinflammatory Mediators
   6 through Downregulation of PI3K/P38 MAPK and NF-κB Signaling in Murine
   7 Macrophages.” *DNA and Cell Biology* 34 (2): 133–41.
   8 Jantan, Ibrahim, Waqas Ahmad, and Syed Nasir Abbas Bukhari. 2015. “Plant-Derived
   9 Immunomodulators: An Insight on Their Preclinical Evaluation and Clinical Trials.”
   10 *Frontiers in Plant Science* 6: 655.
   11 Jr, Charles A. Janeway, Paul Travers, Mark Walport, Mark J. Shlomchik, Charles A. Janeway Jr,
   12 Paul Travers, Mark Walport, and Mark J. Shlomchik. 2001. *Immunobiology*. 5th ed.
   13 Garland Science.
   14 Kantar, Michael B, Justin E Anderson, Sarah A Lucht, Kristin Mercer, Vivian Bernau, Kyle A
   15 Case, Nina C Le, Matthew K Frederiksen, Haley C DeKeyser, and Zen-Zi Wong. 2016.
   16 “Vitamin Variation in Capsicum Spp. Provides Opportunities to Improve Nutritional
   17 Value of Human Diets.” *PLoS One* 11 (8): e0161464.
   18 Kumar, Manish, Subodh Kumar, and Satwinderjeet Kaur. 2011. “Investigations on DNA
   19 Protective and Antioxidant Potential of Chloroform and Ethyl Acetate Fractions of
   20 Koelreuteria Paniculata Laxm.” *Afr J Pharm Pharmacol* 5 (3): 421–27.
   21 Kumar, Vinay, Melissa Lemos, Mansi Sharma, and Varsha Shriram. 2013. “Antioxidant and
   22 DNA Damage Protecting Activities of Eulophia Nuda Lindl.” *Free Radicals and*
   23 *Antioxidants* 3 (2): 55–60.
   24 Lee, K‐J, K Dabrowski, JH Blom, SC Bai, and PC Stromberg. 2002. “A Mixture of Cottonseed
   25 Meal, Soybean Meal and Animal Byproduct Mixture as a Fish Meal Substitute: Growth
   26 and Tissue Gossypol Enantiomer in Juvenile Rainbow Trout (Oncorhynchus Mykiss).”
   27 *Journal of Animal Physiology and Animal Nutrition* 86 (7‐8): 201–13.
   28 Leon, Lisa R, Andrew A White, and Matthew J Kluger. 1998. “Role of IL-6 and TNF in
   29 Thermoregulation and Survival during Sepsis in Mice.” *American Journal of Physiology-*
   30 *Regulatory, Integrative and Comparative Physiology* 275 (1): R269–77.
   31 Lin, Derong, Mengshi Xiao, Jingjing Zhao, Zhuohao Li, Baoshan Xing, Xindan Li, Maozhu
   32 Kong, Liangyu Li, Qing Zhang, and Yaowen Liu. 2016. “An Overview of Plant Phenolic
   33 Compounds and Their Importance in Human Nutrition and Management of Type 2
   34 Diabetes.” *Molecules* 21 (10): 1374.
   35 Macho, Antonio, Marco A Calzado, Juan Muñoz-Blanco, Consuelo Gómez-Díaz, Consuelo
   36 Gajate, Faustino Mollinedo, Plácido Navas, and Eduardo Muñoz. 1999. “Selective
   37 Induction of Apoptosis by Capsaicin in Transformed Cells: The Role of Reactive Oxygen
   38 Species and Calcium.” *Cell Death & Differentiation* 6 (2): 155–65.
   39 Malireddy, Smitha, Sainath R. Kotha, Jordan D. Secor, Travis O. Gurney, Jamie L. Abbott,
   40 Gautam Maulik, Krishna R. Maddipati, and Narasimham L. Parinandi. 2012.
   41 “Phytochemical Antioxidants Modulate Mammalian Cellular Epigenome: Implications in
   42 Health and Disease.” *Antioxidants & Redox Signaling* 17 (2): 327–39.
   43 https://doi.org/10.1089/ars.2012.4600.
   44 Mediratta, PK, KK Sharma, and Surender Singh. 2002. “Evaluation of Immunomodulatory
   45 Potential of Ocimum Sanctum Seed Oil and Its Possible Mechanism of Action.” *Journal*
   46 *of Ethnopharmacology* 80 (1): 15–20.
      1 Mora, J Rodrigo, Makoto Iwata, and Ulrich H Von Andrian. 2008. “Vitamin Effects on the
      2 Immune System: Vitamins A and D Take Centre Stage.” *Nature Reviews Immunology* 8
      3 (9): 685–98.
      4 Nfambi, Joshua, Godfrey S Bbosa, Lawrence Fred Sembajwe, James Gakunga, and Josephine N
      5 Kasolo. 2015. “Immunomodulatory Activity of Methanolic Leaf Extract of Moringa
      6 Oleifera in Wistar Albino Rats.” *Journal of Basic and Clinical Physiology and*
      7 *Pharmacology* 26 (6): 603–11.
      8 Pan, Min‐Hsiung, Geetha Ghai, and Chi‐Tang Ho. 2008. “Food Bioactives, Apoptosis, and
      9 Cancer.” *Molecular Nutrition & Food Research* 52 (1): 43–52.
      10 Poorna, C Asha, MS Resmi, and EV Soniya. 2013. “In Vitro Antioxidant Analysis and the DNA
      11 Damage Protective Activity of Leaf Extract of the Excoecaria Agallocha Linn Mangrove
      12 Plant.” *Agricultural Chemistry. New York: InTech*, 155–66.
      13 Pradhan, D, PK Panda, and G Tripathy. 2009. “Evaluation of the Immunomodulatory Activity of
      14 the Methanolic Extract of Couroupita Guianensis Aubl. Flowers in Rats.”
      15 Prasad, V, V Jain, and AK Dorle. 2010. “Evaluation of Momordica Charantia Ghrita for
      16 Immunomodulatory Activity.” *Journal of Plant Sciences* 5 (1): 99–104.
      17 Rajan, TV. 2003. “The Gell–Coombs Classification of Hypersensitivity Reactions: A Re-
      18 Interpretation.” *TRENDS in Immunology* 24 (7): 376–79.
      19 Reddy, Vanitha, Asna Urooj, and Anila Kumar. 2005. “Evaluation of Antioxidant Activity of
      20 Some Plant Extracts and Their Application in Biscuits.” *Food Chemistry* 90 (1–2): 317–
      21 21.
      22 Santangelo, Carmela, Rosaria Varì, Beatrice Scazzocchio, Roberta Di Benedetto, Carmela Filesi,
      23 and Roberta Masella. 2007. “Polyphenols, Intracellular Signalling and Inflammation.”
      24 *Annali Dell’Istituto Superiore Di Sanita* 43 (4): 394–405.
      25 Schijns, Virgil, Alberto Fernández‐Tejada, Žarko Barjaktarović, Ilias Bouzalas, Jens Brimnes,
      26 Sergey Chernysh, Sveinbjorn Gizurarson, Ihsan Gursel, Žiga Jakopin, and Maria
      27 Lawrenz. 2020. “Modulation of Immune Responses Using Adjuvants to Facilitate
      28 Therapeutic Vaccination.” *Immunological Reviews* 296 (1): 169–90.
      29 Thangaraj, Parimelazhagan. 2019. *Pharmacological Assays of Plant-Based Natural Products*.
      30 Springer.
      31 Valdés-González, Jose Antonio, Marta Sánchez, Ignacio Moratilla-Rivera, Irene Iglesias, and
      32 María Pilar Gómez-Serranillos. 2023. “Immunomodulatory, Anti-Inflammatory, and
      33 Anti-Cancer Properties of Ginseng: A Pharmacological Update.” *Molecules* 28 (9): 3863.
      34 Xu, Xiaolong, Hongquan Li, Xiaolin Hou, Deyin Li, Shasha He, Changrong Wan, Peng Yin,
      35 Mingjiang Liu, Fenghua Liu, and Jianqin Xu. 2015. “Punicalagin Induces Nrf2/HO-1
      36 Expression via Upregulation of PI3K/AKT Pathway and Inhibits LPS-Induced Oxidative
      37 Stress in RAW264. 7 Macrophages.” *Mediators of Inflammation* 2015.
      38 Yahfoufi, Nour, Nawal Alsadi, Majed Jambi, and Chantal Matar. 2018. “The
      39 Immunomodulatory and Anti-Inflammatory Role of Polyphenols.” *Nutrients* 10 (11):
      40 1618. https://doi.org/10.3390/nu10111618.
      41 Yuan, Haidan, Qianqian Ma, Li Ye, and Guangchun Piao. 2016. “The Traditional Medicine and
      42 Modern Medicine from Natural Products.” *Molecules* 21 (5): 559.
      43 Zhang, Bin, Junichi Hirahashi, Xavier Cullere, and Tanya N Mayadas. 2003. “Elucidation of
      44 Molecular Events Leading to Neutrophil Apoptosis Following Phagocytosis: Cross-Talk
      45 between Caspase 8, Reactive Oxygen Species, and MAPK/ERK Activation.” *Journal of*
      46 *Biological Chemistry* 278 (31): 28443–54.
         1 Zuo, Li, Evan R Prather, Mykola Stetskiv, Davis E Garrison, James R Meade, Timotheus I
         2 Peace, and Tingyang Zhou. 2019. “Inflammaging and Oxidative Stress in Human
         3 Diseases: From Molecular Mechanisms to Novel Treatments.” *International Journal of*
         4 *Molecular Sciences* 20 (18): 4472.

5

6

7

8

9

10

11

12

13

14

15

16

17

18

19

20

21

22

23

24

25

26

27

# 28 Table 1 Forward and Reverse Primer of involved in inflammation.

TNF-α	Forward: ACG GCA TGG ATC TCA AAG AC

Reverse: GGT CAC TGT CCC AGC TT

iNOS	Forward: GTG GTG ACA AGC ACA TTT GG Reverse: GGC TGG ACT TTT CAC TCT GC

IL-1β	Forward: GAG TGT GGA TCC CAA GCA AT Reverse: CTC AGT GCA GGC TAT GAC CA

IL-6	Forward: AGT TGC CTT CTT GGG ACT GA Reverse: GCC ACT CCT TCT GTG ACT CC

COX-2	Forward: TCC TCC TGG AAC ATG GAC TC Reverse: TGA TGG TGG CTG TTT TGG TA

GAPDH	Forward: TGC TCG AGA TGT CAT GAA GG Reverse: TTG CGC TCA TCG TAG GCT T

1

2

3

4

5

6

7

8

9

10

11

12

13

14 **Table 2.** Effects of extracts on body weight

| **Groups** | **Initial weight (G)** | **Final weight (G)** | **Weight change** |
| --- | --- | --- | --- |
| **NC** | 100 ± 6.72 | 152.66 ± 7.91 | 52.66 ± 0.84 |
| **DC** | 101 ± 5.44 | 133.5 ± 2.66^**^ | 32.5 ± 1.96 |
| **ML** | 103.16 ± 8.01 | 166.16 ± 7.13 | 63.00 ± 0.62 |
| **MLMExCC100** | 101 ± 8.53 | 144.66 ± 10.69 | 43.66 ± 1.53 |
| **MLMExCC200** | 104.66 ± 5.39 | 144.16 ± 8.01 | 39.5 ± 1.85 |
| **MExCC100** | 109.33 ± 6.74 | 163.16 ± 8.88 | 53.83 ± 1.51 |
| **MExCC200** | 107.5 ± 8.09 | 133.33 ± 11.03^**^ | 25.83 ± 2.08 |

1 Values are expressed as mean ± SD, where *n* = 6 animals were compared with one-way ANOVA,
2 followed by Tukey’s multiple comparison test. ***p* < 0.01.

3

4

5

6

7

8

9

10

11

12

13

14

# 15 Table 3 Effects of extracts on organs weight

| **Groups** | **Liver weight(G)** | **Spleen weight(G)** | **Thymus weight(G)** |
| --- | --- | --- | --- |
| **NC** | 5.98 ± 0.42 | 0.47 ± 0.02 | 0.47 ± 0.04 |
| **DC** | 6.60 ± 0.35 | 0.56 ± 0.06 | 0.4 ± 0.03 |
| **ML** | 6.14 ± 0.18 | 0.51 ± 0.04 | 0.5 ± 0.40 |
| **MLMExCC100** | 6.00 ± 0.23 | 0.04 ± 0.45 | 0.39 ± 0.03 |
| **MLMExCC200** | 5.66 ± 0.22 | 0.46 ± 0.05 | 0.37 ± 0.02 |
| **MExCC100** | 6.04 ± 0.44 | 0.47 ± 0.03 | 0.44 ± 0.08 |
| **MExCC200** | 6.61 ± 0.64 | 0.51 ± 0.06 | 0.23 ± 0.043^***^ |

1 Values are expressed as mean ± SD, where *n* = 6 animals were compared with one-way ANOVA,
2 followed by Tukey’s multiple comparison test. ****p* < 0.001.

3

4

5

6

7

8

9

10

11

12

13

14

# 15 Figure Legends

1 **Figure 1**. The effects of *C. chinense* extracts on the delayed type of hypersensitivity of
2 experimental animals (n=6). Animals were fed with ML, MLMExCC and MExCC develop
3 hypersensitivity. Values are expressed as mean ± SD for six animals in each group compared with
4 Two-way ANOVA followed by Tukey’s multiple comparison test.
5 **Figure 2. A**. Total count **B**. Differential counts of WBCs are assessed. Comparisons were made
6 with normal control. Inference (Two way ANOVA with Tukey’s multiple comparison) to interpret
7 significant difference. Data expressed as mean ± S.D where n = 6. *= p < 0.05 **= p < 0.01.
8 ns=nonsignificant.
9 **Figure 3.** Serum albumin/ globulin ratio are assessed. Comparisons were made with normal
10 control. Inference (Two-way ANOVA with Tukey’s multiple comparison) to interpret significant
11 differences. Data expressed as mean ± S.D where n = 6. **= p < 0.01, ns = nonsignificant.
12 **Figure 4.** Image of the spleen tissues from the experimental animal groups as seen through
13 histopathology. The white pulp and red pulp spleen cell are seen in the image (microscopic
14 resolution: 10 × 40). Hematoxylin and eosin‐stained rat spleen micrographs. The images displayed
15 are the glomerulus slices counterstained with hematoxylin and stained with PAS.
16 **Figure 5.** Image of the thymus tissues from the experimental animal groups as seen through
17 histopathology. The cortex and medulla of thymus are seen in the image (microscopic resolution:
18 10 × 40). Hematoxylin and eosin‐stained rat thymus micrographs. The images displayed are the
19 glomerulus slices counterstained with hematoxylin and stained with PAS.
20 **Figure 6.** Effects of PEExCC, EAExCC and MExCC on the expression of pro-inflammatory
21 cytokines in LPS-stimulated RAW 264.7 macrophages. The expression of (A) cyclooxygenase-2
22 (COX-2) (B) inducible nitric oxide synthase (iNOS) (C) IL-1β, (D) IL-6 and TNF-α. Values
23 represent the mean ± SD. Statistical significance was calculated by *t*-test and one-way ANOVA. *
24 *p* < 0.05, ** *p* < 0.01, *** *p* < 0.001 indicate significant differences compared to the LPS.
25 **Figure 7.** DNA damage protecting effects of A. PEExCC; B. EAExCC and C. MEExCC.
26 Electrophoretic pattern of pBR322 supercoiled plasmid DNA after treatment of Fenton’s reagent
27 followed by different concentrations. R = single-stranded relaxed nicked DNA (R-Form), L=
28 double-stranded nicked and linear DNA (L-Form), C = native circular DNA (scDNA). For
29 electrophoresis, 1% gel was used with 10 mg/mL ethidium bromide and running in 1x TBE buffer
30 (pH 8.2) at 90 V for 90 min. Lane-1- Untreated plasmid DNA, Lane-2-Treated with Fenton’s
31 reagent without extract and Lane 3-7.
1 **Figure 8. A.** Pictoral view of cytotoxic activity of EAExCC, PEExCC and MExCC in Vero and
2 BHK 21 cells. **B**. Pictoral view of cytotoxic activity of EAExCC, PEExCC and MExCC in HeLa
3 cells.

4

5

6

7

8

9

10

11

12

13

14

15

16

17

18

19

20

21

22

23

24

25

26

100

NC DC

ML

MLMExCC100 MLMExCC200

MExCC100

MExCC200

a

a

a

b

b

**0 h**

**4 h**

**8 h**

**24 h**

80

% increment in food pad thickness

60

40

20

0

-20

1

2 **Figure 1**. The effects of *C. chinense* extracts on the delayed-type hypersensitivity of experimental
3 animals (n=6). Animals fed with ML, MLMExCC and MExCC develop hypersensitivity. Values
4 are expressed as mean ± SD for six animals in each group compared with Two-way ANOVA
5 followed by Tukey’s multiple comparison test. a=p <0.05 and b = *p* < 0.001 compared with the
6 normal control animals (significantly different).

7

8

9

10

11

12

13

1

^2^	**A.**

3

4

**No of WBC/μL**

5

6

7

8

9

10

11

# B.

12

13

14

15

16

17

18

19

20

21

22

10000

8000

ns

✱✱

6000

4000

2000

0

**80**

✱

ns

✱✱

**60**

**No of WBCs(%)**

**40**

**20**

**0**

**NC**

**DC**

**ML**

**MLMEx100**

**MLMEx200**

Lymphocyte Neutrophils

NC

DC

ML

MLMExCC100

MLMExCC200

MExCC100

Eosinophil Monocyte

MExCC200

23 **Figure 2.** A. Total counts and B. Differential counts of WBCs are assessed. Comparisons were

**MEx100**

**MExCC200**

24 made with normal control. Inference (Two-way ANOVA with Tukey’s multiple comparison) to
25 interpret significant differences. Data expressed as mean ± S.D where n = 6. *= p < 0.05 **= p <
26 0.01. ns= nonsignificant

27

1

NC DC

MLMExCC100

ML MLMExCC200 MExCC100

MExCC200

8	8

ns

✱✱

✱

6	6

Albumim/Globulin

Serum Protein Values(mg/dL)

4	4

2	2

0	0

Serum Protein

AGR

Serum Protein

AGR

Serum Protein

AGR

Serum Protein

AGR

Serum Protein

AGR

Serum Protein

AGR

Serum Protein

AGR

2 F**igure 3.** Serum albumin/ globulin ratio are assessed. Comparisons were made with normal
3 control. Inference (Two-way ANOVA with Tukey’s multiple comparison) to interpret significant
4 differences. Data expressed as mean ± S.D where n = 6. **= p < 0.01, ns = nonsignificant.

5

6

7

8

9

10

11

12

13

14

15

1

2

3

4

6

7

8

9

**A**

**B**

**D**

5

**C**

**E**

**F**

14

10

11

12

13

**G**

15

16

17

18

19

20 **Figure 4.** Image of the spleen tissues from the experimental animal groups as seen through
21 histopathology. The white pulp and red pulp spleen cell are seen in the image (microscopic
22 resolution: 10 × 40). Hematoxylin and eosin‐stained rat spleen micrographs. The images displayed
23 are the glomerulus slices counterstained with hematoxylin and stained with PAS.

24

25

26

27

1

2

3

4

**A**

**B**

5

6

7

8

9

10

11

12

13

**C**

**D**

**G**

**E**

**F**

14

15

16

18

17

19 **FIGURE 5.** Image of the thymus tissues from the experimental animal groups as seen through
20 histopathology. The cortex and medulla of thymus are seen in the image (microscopic resolution:
21 10 × 40). Hematoxylin and eosin‐stained rat thymus micrographs. The images displayed are the
22 glomerulus slices counterstained with hematoxylin and stained with PAS.

23

**COX-2**

**10**

ns

✱✱

✱✱

✱✱✱

Relative mRNA expression (fold change)

**iNOS**

**4**

ns

ns

✱

✱✱✱

Relative mRNA expression (fold change)

**8**

**3**

**6**

**2**

**4**

**1**

**2**

**_0_	0**

Control

LPS

PEExCC

EAExCC

MExCC

Control

LPS

PEExCC

EAExCC

MExCC

**2.0**

Relative mRNA expression (fold change)

**1.5**

**1.0**

**0.5**

**0.0**

2

3

4

5

6

7

**IL-1β**

**IL-1**β

✱✱✱

✱✱✱

✱✱✱

✱✱✱

 Control PEExCC EAExCC

 MExCC LPS

**4**

ns

ns

✱✱

✱✱✱

Relative mRNA expression (fold change)

**3**

**2**

**1**

**0**

**Control**

**LPS**

**PEExCC**

**EAExCC**

**TNF-α**

**IL-6**

**6**

✱✱✱ ✱✱✱

✱✱✱

✱✱✱

Relative mRNA expression (fold change)

**4**

**2**

**0**

IL-6 IL-6 IL-6 IL-6 IL-6

1

Control LPS PEExCC EAExCC MExCC

8

**MExCC**

9 **Figure 6.** Effects of PEExCC, EAExCC and MExCC on the expression of pro-inflammatory
10 cytokines in LPS-stimulated RAW 264.7 macrophages. The expression of (A) cyclooxygenase-2
11 (COX-2) (B) inducible nitric oxide synthase (iNOS) (C) IL-1β, (D) IL-6 and TNF-α. Values
12 represent the mean ± SD. Statistical significance was calculated by *t*-test and one-way ANOVA. *
13 *p* < 0.05, ** *p* < 0.01, *** *p* < 0.001 indicate significant differences compared to the LPS.

1

B.

A.

^2^	C.

3

4

5

6

7

8

9

10

11 **Figure 7** DNA damage protecting effects of A. PEExCC; B. EAExCC and C. MEExCC.
12 Electrophoretic pattern of pBR322 supercoiled plasmid DNA after treatment of Fenton’s reagent
13 followed by different concentrations. R = single-stranded relaxed nicked DNA (R-Form), L=
14 double-stranded nicked and linear DNA (L-Form), C = native circular DNA (scDNA). For
15 electrophoresis, 1% gel was used with 10 mg/mL ethidium bromide and running in 1x TBE buffer
16 (pH 8.2) at 90 V for 90 min. Lane-1- Untreated plasmid DNA, Lane-2-Treated with Fenton’s
17 reagent without extract and Lane 3-7.

18

1

2

3

4

5

6

7

8

9

10

11

12

13

14

15

16

17

18

19

20

21

**PE + BHK 21**

**EA + Vero**

**EAExCC +Vero**

**PE +Vero**

**PEExCC+ Vero**

**M +Vero**

**MExCC +Vero**

**EA + BHK 21**

**EAExCC + BHK 21**

27	A.

23

24

25

26

**PEExCC + BHK 21**

22

**MExCC + BHK 21**

**M + BHK 21**

1

2

3

4

5

6

7

8

9

**EA + HeLa**

**EAExCC + HeLa**

**PE + HeLa**

**PEExCC +HeLa**

**M + HeLa**

**MExCC + HeLa**

# 14 B.

15 **Figure 8. A.** Pictoral view of cytotoxic activity of EAExCC, PEExCC and MExCC in Vero and

10

11

12

13

16 BHK 21 cells. **B**. Pictoral view of cytotoxic activity of EAExCC, PEExCC and MExCC in HeLa
17 cells.

18

19
