# Supplementary material for: Capsicum chinense Jacq. fruit plays an immunomodulatory role in cytokine attenuation and DNA damage protection
Source: PLoS One. 2025 Feb 25;20(2):e0319251. doi: 10.1371/journal.pone.0319251 (PMC11856283; doi:10.1371/journal.pone.0319251)
Supplement: Attachment — Submitted filename: Response to Editorial Comments2.docx [file pone.0319251.s005.docx]

Dr. MD. ATIAR RAHMAN

MSc (University of Dhaka), PhD (Ehime University, Japan), Post-Doc (University of KwaZulu Natal, South Africa)

**Dean,**

**Faculty of Biological Sciences,**

**University of Chittagong**

**and Professor**

Department of Biochemistry and Molecular Biology

**University of Chittagong**

Chittagong-4331, Bangladesh

Fellow, Bangladesh Academy of Sciences

Dept website: [www.bmb.cu.ac.bd](http://www.bmb.cu.ac.bd)

Laboratory UR: www.am-npr.cu.ac.bd

W. ‡gvt AvwZqvi ingvb

**Aa¨vcK**

cÖvYimvqb I AbycÖvY weÁvb wefvM

PÆMÖvg wek¦we`¨vjq, PÆMÖvg-4331, evsjv‡`k

‡dvb:wcGweG· +880-31-726311-14, 2606001-10, G·‡Ubkb:4334

d¨v·: +880-31-726310

E-mail: [atiar@cu.ac.bd](mailto:atiar@cu.ac.bd)

Website: [www.cu.ac.bd](http://www.cu.ac.bd)

Cell: +660971174180

Date: 10 October 2024

To

The Editor

Plos One

#### **Dear Editor**

Thank you so much for your comments on our revised version of the manuscript entitled “***Capsicum chinense Jacq. fruit plays an immunomodulatory role in a cytokine attenuation and DNA damage protection***” to your esteemed journal “**Plos One**”. We have gone through the comments your comments and responded in a point-to-point manner as follows:

**Editorial comments:**

**1. Please amend your list of authors on the manuscript to ensure that each author is linked to an affiliation.**

**We note that you have included affiliation numbers 1 and 2 however only affiliations 1 have authors linked to them. Please amend affiliation 2 to link an author to it or remove if added in error.**

**Author’s Response: Thank you so much for bringing the issue to the attention of the authors. Authors have addressed the issue and removed the affiliation 2.**

Md Atiar Rahman PhD

Corresponding author and

Professor

Department of Biochemistry and Molecular Biology

University of Chittagong

Bangladesh

**Fellow, Bangladesh Academy of Sciences**

**ORCID**: https://orcid.org/0000-0002-4902-8923

**Researcher ID**: https://publons.com/researcher/362002/rahman-md-atiar/

**Scopus ID**: https://www.scopus.com/authid/detail.uri?authorId=55457954600

**LiveDNA**: [https://livedna.org/880.19039](https://livedna.org/880.19039)

## *Institutional Webpage*: *www.cu.ac.bd*

**Personal Webpage**: **www.am-npr.cu.ac.bd**

## *Google scholar ID: https://scholar.google.com/citations?hl=en&user=Hrbt-8QAAAAJ*
