# Supplementary material for: Capsicum chinense Jacq. fruit plays an immunomodulatory role in cytokine attenuation and DNA damage protection
Source: PLoS One. 2025 Feb 25;20(2):e0319251. doi: 10.1371/journal.pone.0319251 (PMC11856283; doi:10.1371/journal.pone.0319251)
Supplement: Attachment — Submitted filename: Response to Reviewers.docx [file pone.0319251.s007.docx]

Dear Editor

Thank you so much for your time and patience to leave the comments on our revised version of the manuscript entitled “***Capsicum chinense Jacq. fruit plays an immunomodulatory role in a cytokine attenuation and DNA damage protection***” to your esteemed journal “**Plos One**”. We have gone through the comments your comments and responded in a point-to-point manner as follows:

**The collection of fruit and verification of its authenticity gives rise to certain concerns. The fruit was obtained from the farmers market. By what means was the fruit determined to be free from any adulteration?**

**Dear editor,**

Thank you so much to repeat the questions. Let us answer the questions’ part by part”

**Editor’s query: The collection of fruit and verification of its authenticity gives rise to certain concerns. The fruit was obtained from the farmer’s market.**

**Author’s response:** The fruits were collected from the farmers market, however; the quality of fruits was ensured to be free from adulteration. The major criteria were the colour, appearance, texture, flavour, weight, and volume. No spotted/rotten/unhealthy/infectious fruits were accepted. Very fresh and natural fruits were accepted for our research.

**Based on what criteria was the botanical number assigned?**

The fruit was identified and botanically assigned by Dr. Shaikh Bokhtear Uddin ([bokhtear@cu.ac.bd](mailto:bokhtear@cu.ac.bd)) who is a plant taxonomist and Professor of the department of Botany, University of Chittagong. However, the following approaches were maintained to prepare and taxonomically identify the sample.

Plant description was made including the plant habit (tree, shrub, vine, herb), height, growth form, color, bark, branching, leaf orientation, or general volume/size/spread of a plant. Detailed location of the plant, origin of material, habitat, frequency (whether the plant is rare or common), herbarium sheet preparation (pressing and drying the sample, labelling, mounting), comparing with the published plant descriptions, illustrations and photographs to identify the herbarium specimens.

**The experimental design included the use of milk in some groups. What was the rationale for using milk?**

The capsaicinoids of *C. chinense* generally accounts for their pungency. Capsaicinoids irritate the stomach lining and lead to an increase in stomach acid production. Milk protein facilitates an emulsification during gastrointestinal digestion. Therefore, milk was used to extensively reduce the pungency of capsaicinoids produced by *C. chinense*.

**Please specify the origin and nutritional composition of the milk. Please specify that you have standardized the milk composition.**

Milk was collected from Bangladesh Standards and Testing Institution (BSTI) recognized standard commercial brand, Arong, which is one of the leading brands in Bangladesh. The collected milk is already standardized by BSTI, and we ensured the standardization. The nutritional composition of milk is as follows:

| Fat percentage | 3.778 ± 0.018 |
| --- | --- |
| Protein percentage | 3.282 ± 0.016 |
| Lactose percentage | 4.398 ± 0.045 |
| SNF percentage | 8.345 ± 0.028 |
| Density percentage | 1.024 ± 0.001 |
| Mineral percentage | 0.718 ± 0.019 |
| Freezing point | 0.4773 ± 0.004 |
| Specific gravity | 1.029 ± 0.0060 |

**Please specify the in-vitro and in-vivo procedures separately?**

Thank you so much for your suggestion. In fact, the in vivo and ain vitro procedures are presented separately.

The In vivo Immunomodulating activity of *C. chinense* Jacq, with Long Evans rats and follow-up experimentations (immunization, Body weight and lymphoid organ weight, total serum protein and albumin: globulin ratio, total leukocyte count, differential count etc) whereas in vitro experimentations (Anti-Inflammatory activity against LPS-stimulated RAW264.7 macrophages) were carried out with follow-up experimentation.
