# Supplementary material for: Capsicum chinense Jacq. fruit plays an immunomodulatory role in cytokine attenuation and DNA damage protection
Source: PLoS One. 2025 Feb 25;20(2):e0319251. doi: 10.1371/journal.pone.0319251 (PMC11856283; doi:10.1371/journal.pone.0319251)
Supplement: Attachment — Submitted filename: Response to reviewers.docx [file pone.0319251.s009.docx]

**Reviewer #3: Review Report:**

**The manuscript titled "Capsicum chinense Jacq. fruit plays an immunomodulatory role in a cytokine attenuation and DNA damage protection" suggests that Capsicum chinense Jacq. fruit has potential health benefits by modulating the immune system. It implies the fruit can reduce the overproduction of cytokines, which are signaling proteins involved in inflammation. Additionally, the fruit may protect against DNA damage, highlighting its role in cellular protection and overall immune health. Overall, the manuscript appears to present a promising study with implications for both basic research and clinical practice which highlights its potential as a natural therapeutic agent for enhancing immune health and cellular resilience.**

**Note: I reviewed the file named (PONE-D-24-29500-R3) downloaded from journal online portal and all my comments are according to the line numberings of the pdf file “PONE-D-24-29500-R3”**

**Detailed Comments/Recommendations:**

**Title:**
**¬The title of the study, "Capsicum chinense Jacq. fruit plays an immunomodulatory role in a cytokine attenuation and DNA damage protection" appears to be apt and engaging. It effectively highlights the fruit's potential therapeutic significance, appealing to researchers and healthcare professionals interested in natural interventions for inflammation and cellular protection.**

**Abstract:**
**¬It will be more attractive for the readers if the authors may add up aims and objective of the study at the start of abstract in 2 lines.**

Author’s Response - We sincerely appreciate your diligence in reviewing our manuscript. The authors have outlined the aims and objectives at the outset of the document abstract.
 **¬Page 4, line 3: Mention the animal (rats).**

Author’s Response – We appreciate your feedback. The authors have referenced the rats’ species (Long Evans rats) utilized in the study.

**¬Page 4, Line 6: Mention the dose at which immune response was suppressed.**

Author’s Response- Thank you so much for your thoughtful review of our manuscript! We're happy to share that we've included information about the dose at which the immune response was suppressed.

**¬It will be more appropriate if the authors may add a few numerical results in the abstract. It will be more impressive for readers.**

Author’s Response: We sincerely appreciate your insightful comments. The authors have incorporated numerical results into the abstract, which will enhance its impact for the readers.
**¬Key Words: Well written.**

Author’s Response -Thank you so much.

**Introduction:**
**¬Page 5, Lines 8-13: Sentence is too long; it will be more impressive if authors may split into portions for the better understandings of the reader.**

Author’s Response: We sincerely appreciate your valuable feedback. We have divided the sentence into segments to enhance the reader's understanding.

**¬Page 5, Line 15-18: The statement is confusing for the reader. It can be rewritten for the ease of reader.**

Author’s Response: We sincerely appreciate your feedback. The authors have revised the statement to eliminate any potential confusion for the readers.

**¬Page 6, Lines 4 to onward: Please add** **biography of Capsicum chinense Jacq, along with its habitat and cultivations etc.**

Author’s Response- Thank you so much for your suggestion. In response, we have included the biography of *Capsicum chinense* Jacq.

**Materials and Methods:**

**¬Page 7, Line 8: How much Capsicum chinense Jacq. Was obtained? Please mention its quantity.**

Author’s Response- We extend my sincere gratitude for your invaluable observation and for the manner in which the authors have addressed your suggestions.

**¬Page 8, Line 6: Please mention temperature and pressure adjusted in rotary evaporator.**

Author’s Response- Much appreciated. We mentioned the temperature and pressure adjustments that occurred in the rotary evaporator.

**¬Page 8, Line 18: Kindly mention humidity conditions, provided to animals in animal house.**

Author’s Response- Thank you for your thoughtful reply! The authors highlighted the humidity conditions in the animals' house.

**¬Page 8, Line 3: Please mention quantity of antigen administered to animals along with route of administration.**

Author’s Response-We truly appreciate your keen observation! We're excited to let you know that we have taken your feedback into account and made the necessary adjustments.

**¬Page 10, Line 3: Please insert reference for the methodology of immunization.**

Author’s Response- In response to your suggestion, we happily included the reference for the methodology of immunization.
**¬Pages 10-11: References are missing for all the methods mentioned on page 10 and 11.**

Author’s Response-We’ve added the references just like you suggested!
**¬ Page 11, Line 20: Please mention magnification/resolution power at which slides were observed under microscope.**

Author’s Response- We would like to express our sincere gratitude. The authors have indicated the resolution capacity of the microscope.
**¬Page 15, Line 4: please insert reference.**

Author’s Response-The authors inserted the reference.
**¬**
**Results:**
**¬Page 16, Line 12-14: Please write the sentence in past tense.**

Author’s Response- We express our gratitude for your insightful observation. The sentence has been composed in the past tense.
**¬All results are well expressed**

**Discussion:**
**¬Page 21, Line 17-18: give full form of bw.**

Author’s Response-Thank you. We have written the full form of bw.
**¬Over all the discussion is well written and quite satisfactory.**

**Conclusion:**
**¬It is well written. The authors are requested to add on future prospects of the study in the last of conclusion.**

Author’s Response- We appreciate your valuable comment. The authors have included the future prospects of the study in the conclusion section.

**References:**
**¬Page 33, Line 7: The page numbers are missing.**

Author’s Response- The page number is included.
**¬The authors are advised to review all the references for the strict adherence of the uniformity of Journal’s references style.**

Author’s Response- Thank you for your guidance. We have thoroughly examined all the references to ensure strict adherence to the Journal’s referencing style.
**Tables:**
**¬Table 2: Please add unit (g) in the column of weight change.**
Author’s Response- Thank you. The authors have added the unit in Table 2.

**Concluding Remarks:**

**The paper necessitates the aforementioned corrections, inclusive of rectifying any errors highlighted, coupled with a meticulous proofreading to refine its English language usage. Upon addressing these amendments, the paper will meet the required standards for acceptance.**

**Reviewer #4: The manuscript entitles as “Capsicum chinense Jacq. fruit plays an immunomodulatory role in a cytokine attenuation” is written by Saha et al., is on the role of Capsicum chinense’s fruits in cytokine attenuation. The manuscript is interesting and can be a new addition to the literature but needs to address some comments before acceptance.**

**1.The graphic abstract needs to be revised by adding the necessary images only to make it more concise and informative.**

Author’s Response: We would like to express our sincere gratitude for your diligent efforts in reviewing our manuscript. We acknowledge the importance of your insightful comments and suggestions, which significantly contribute to the enhancement of our work. The authors have revised the graphical abstract by incorporating the necessary images solely to render it more concise and informative**.**

**2.Did the authors isolate/identified chemical compounds from the study plant’s fruits and screen them for these biological activities. The activity of the extracts can be due to the synergistic effect of the mixture of compounds and pure compounds can be more prominent and effective.**

**Author’s Response:** Thank you so much for your time and patience to review our manuscript. The authors indeed identified chemical compounds by GC-MS from the fruits of the plant and evaluated them for certain biological activities mentioned in another article by the authors. The DOI for that article is <https://doi.org/10.1016/j.jff.2024.106103>. Since most of the compounds exhibit antioxidant, anti-inflammatory, and antimicrobial properties, we can confidently assert that the effects of these extracts are significant and compelling. Regulating immune responses becomes especially important during periods of heightened oxidative stress, which can weaken immune cell activity and lead to a state of systemic inflammation known as oxinflammation. Phytochemicals extracted from fruit, which are high in antioxidants, may help restore the balance between antioxidants and oxidants and modulate the immune response.

**3.Why did the authors select methanol extract and use two doses of high concentrations (100 and 200 mg) only which are not sufficient to see the immunomodulatory effects.**

**Author’s Response:** Thank you so much for your valuable comments. In the author’s preceding article (https://doi.org/10.1016/j.jff.2024.106103), it is unequivocally demonstrated that among the three extracts (petroleum ether, ethyl acetate, and methanol), the methanolic extracts exhibited the most pronounced antioxidative effect and significantly reduced hyperglycemia induced by oxidative stress in comparison to the other two extracts. We chose methanolic extracts for this reason.

We conducted a baseline selection based on an acute toxicity(LD_50_) test to determine the doses, that guided our choice of concentration levels. The test animals received a methanolic extract of *C. chinense* (500–2000 mg/kg). Results showed that the highest dose tested did not lead to mortality.  Moving forward, we will explore other concentration experiments to broaden our efforts.

**4.What molecular pathways are involved in the** **dose-dependent immunomodulatory effects of C. chinense methanol extract?**

**Author’s Response:** We greatly appreciate your insightful feedback. The authors have examined the molecular pathways relevant to the dose-dependent immunomodulatory effects of C. chinense methanol extract in the discussion section of the article. In this section, the authors further clarified the mechanism.

**Phagocytic Activity of Neutrophils**:

The extract augments the phagocytic activity of neutrophils, constituting an integral component of the cell-mediated immune response. This is evidenced by an elevated neutrophil count and their enhanced capacity to adhere and migrate towards pathogens. Such observations signify activating pathways associated with neutrophil recruitment and adhesion, potentially involving cytokines and other chemokines.

**T-Cell Proliferation**:

The extract stimulates T-cells by boosting the delayed-type hypersensitivity (DTH) response. This process probably engages the T-cell receptor (TCR) signaling pathway, which is essential for T-cell activation and cytokine production release.

**Cytokine Release and Inflammatory Response**:

The extract enhances cytokine release, thereby promoting the inflammatory response. This process may engage the nuclear factor-kappa B (NF-κB) and mitogen-activated protein kinase (MAPK) pathways, which govern the expression of cytokines and other immune factor mediators.

**Immunoglobulin Production**:

The extract promotes the production of immunoglobulins, indicating a role in B-cell activation pathways and class-switch recombination, possibly influenced by cytokines such as IL-6.

**Major Histocompatibility Complex (MHC) Expression**:

Amino acids in the extract facilitate the production of MHC complexes, which are crucial for antigen presentation. This process includes pathways such as the antigen-processing and presentation pathways regulated by MHC class I and II molecules.

**Trace Element Utilization**:

The trace elements within the extract support T-cell proliferation and the function of lytic enzymes. They might affect pathways such as the calcineurin-NFAT (nuclear factor of activated T-cells) pathway, which plays a vital role in T-cell activation.

**Amino Acids and Immunomodulation**:

The amino acids in the extract might help produce immunoglobulins and various immune mediators. This relates to protein biosynthesis pathways and their contribution to strengthening the immune response.

In conclusion, the immunomodulatory effects of the methanol extract derived from *C. chinense* are likely mediated through mechanisms that encompass T-cell activation, cytokine release, phagocytosis, immunoglobulin production, and the expression of major histocompatibility complex (MHC). Additionally, the involvement of trace elements and amino acids in facilitating these processes is also noteworthy.

**5.There are so many typographical mistakes in the manuscript and need to be revised by native speaker.**
**6.The references are not properly arranged according to the Journal format and also cross checked with citation in the text.**

**Author’s Response:** Thank you so much for your valuable observation. W addressed your suggestions.

**Reviewer #5: In this study,a number of organic extracts of Capsicum chinense Jacq. (methanol, ethyl acetate and petroleum ether) have been studied for their immunomodulatory and DNAdamage-protective functions. Detailed explanations are provided for the study. The submission needs to be revised before it can be accepted.**

**1.In the materials & methods section, you mentioned defatting the powder sample using n-hexane, petroleum ether, ethyl acetate and methanol. But there is no more detail about this procedure. What ratio did you use those solvents and what is the volume of the reaction vessel? If you used a method of previously published work, I suggest you add a reference to this section.**

**2.The sentence in section 2.4.1 “The animals were placed in polycarbonate transparent rat cages (430 mm long, 290 mm wide, and 201 mm high) maintaining at temperatures ranging from (24 ± 2) ºC and a dark-light cycle lasting 12 h.” should be rewritten.**

**Author’s Response:** I sincerely appreciate your invaluable observations and acknowledge your suggestions. Rewritten.

**3.For overall, which extract has the best immunomodulatory effect methanol extract, ethyl acetate extract or petroleum ether extract?**

**Author’s Response: I genuinely value your insightful observations and appreciate your suggestions. Certainly, methanolic extracts display the most effective immunomodulatory properties.**
