# Supplementary material for: Capsicum chinense Jacq. fruit plays an immunomodulatory role in cytokine attenuation and DNA damage protection
Source: PLoS One. 2025 Feb 25;20(2):e0319251. doi: 10.1371/journal.pone.0319251 (PMC11856283; doi:10.1371/journal.pone.0319251)
Supplement: Attachment — Submitted filename: pone.0319251.docx [file pone.0319251.s011.docx]

**Response to reviewers**

Date: 19 January 2025

To

The Editor

Plos One

#### **Dear Editor**

Thank you so much for your valuable time and patience to handle our manuscript entitled “***Capsicum chinense Jacq. fruit plays an immunomodulatory role in a cytokine attenuation and DNA damage protection***” to your esteemed journal “**Plos One**”. We are indeed very grateful that you put your utmost efforts to improve our manuscript through repetitive review process. We have gone through the comments of the reviewers in this phase and addressed all those through a rigorous revision of the manuscript. Authors’ point-to-point responses are duly included in the submission system.

All the comments have been addressed in the previous revision, however; in this version we have received only the following comment which has been as addressed in the following way:

**Additional Editor Comments:**

**Dear Authors**

**In previous revisions, I repeatedly asked the authors to add the composition of milk in the text. Once more, please include the composition or give a scientific justification for its exclusion; otherwise, the manuscript can be rejected.**

**Author’s response:** Thank you so much for your suggestion. The authors have inserted the composition and source of milk in the discussion section. A clear justification of using the milk is also incorporated in the text. A table is as well added in the table section to detail the milk composition. Hope it will completely quench the queries of the reviewers/editors.

We do hope that the revised manuscript will be led to fall into the next phase of publication procedures.

Your further responses will be highly appreciated.

Md Atiar Rahman PhD

Corresponding author

and

Professor

Department of Biochemistry and Molecular Biology

University of Chittagong

Bangladesh

**Fellow, Bangladesh Academy of Sciences**

**ORCID**: https://orcid.org/0000-0002-4902-8923

**Researcher ID**: https://publons.com/researcher/362002/rahman-md-atiar/

**Scopus ID**: https://www.scopus.com/authid/detail.uri?authorId=55457954600

**LiveDNA**: [https://livedna.org/880.19039](https://livedna.org/880.19039)

## *Institutional Webpage*: *www.cu.ac.bd*

**Personal Webpage**: **www.am-npr.cu.ac.bd**

## *Google scholar ID: https://scholar.google.com/citations?hl=en&user=Hrbt-8QAAAAJ*
